# Supplementary figures and images for: The SARS-CoV-2 accessory protein Orf3a is not an ion channel, but does interact with trafficking proteins
Source: eLife. 2023 Jan 25;12:e84477. doi: 10.7554/eLife.84477 (PMC9910834; doi:10.7554/eLife.84477)

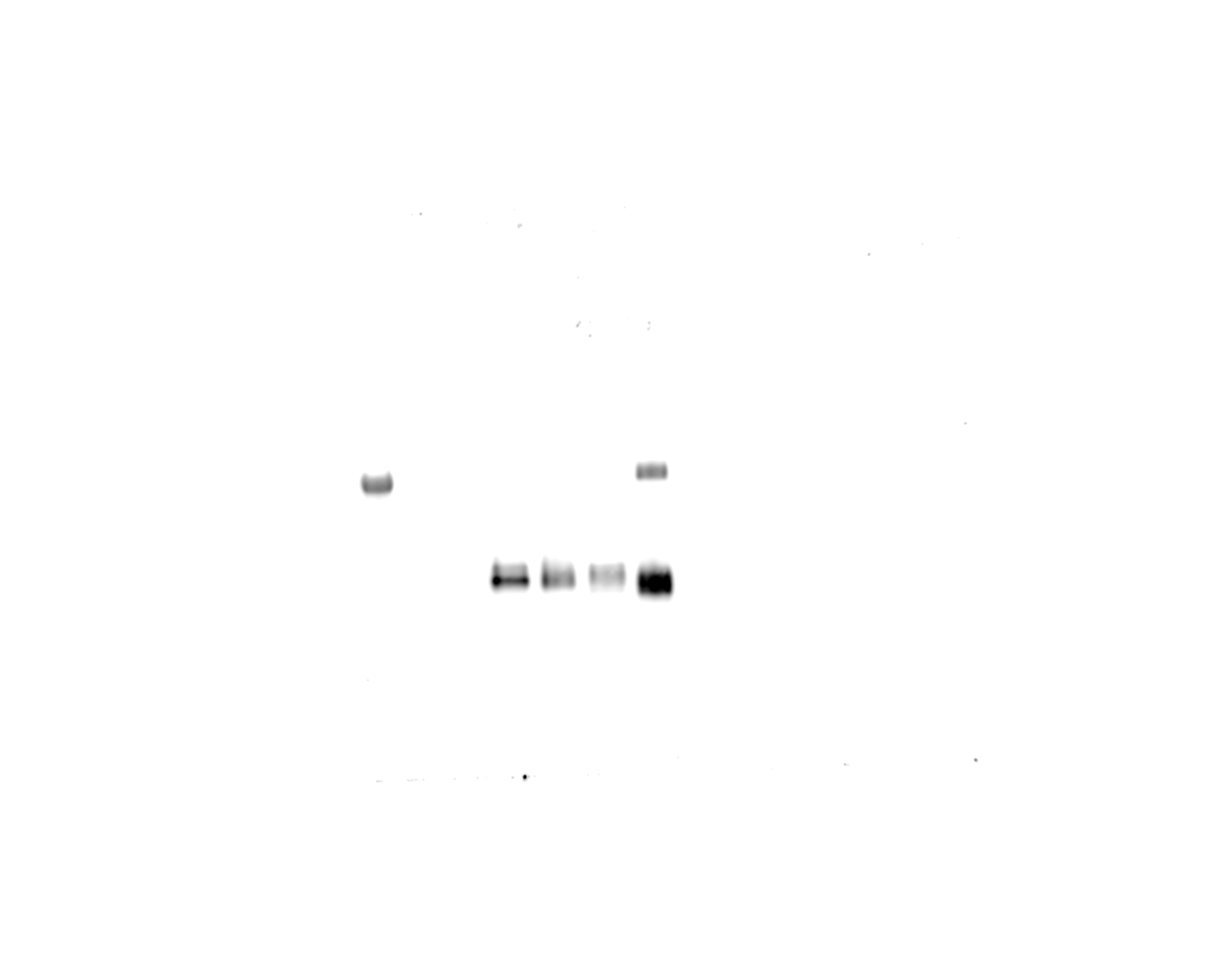

Supplement: Figure 2—figure supplement 3—source data 1. [file elife-84477-fig2-figsupp3-data1.zip › Figure 2-Figure Suppement 3-source data 1/Orf3a_oocyte_biotinylation samples_blot_only.tif]

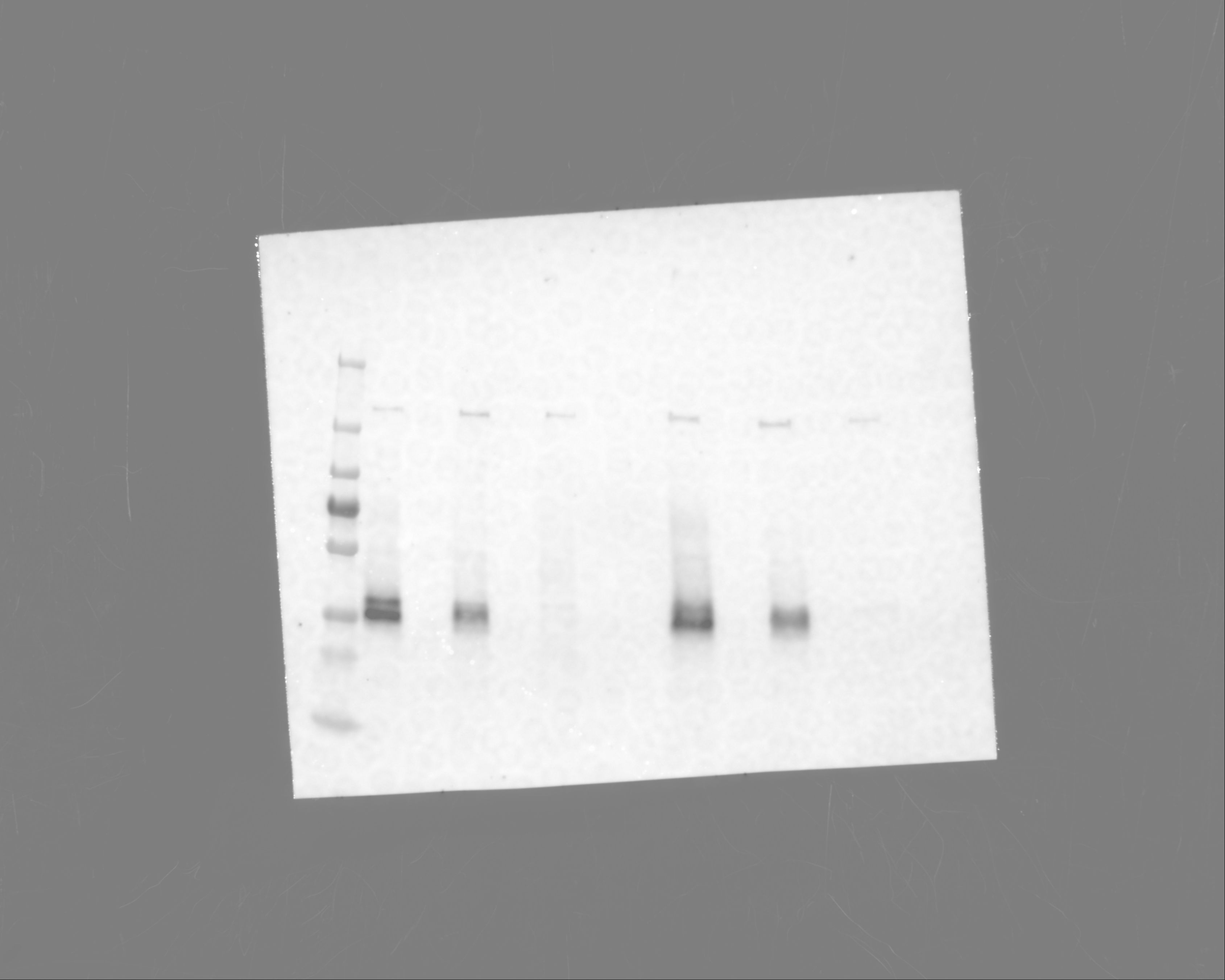

Supplement: Figure 2—figure supplement 3—source data 1. [file elife-84477-fig2-figsupp3-data1.zip › Figure 2-Figure Suppement 3-source data 1/Orf3a_oocyte_no biotinylation reagent_standardsmerged.tif]

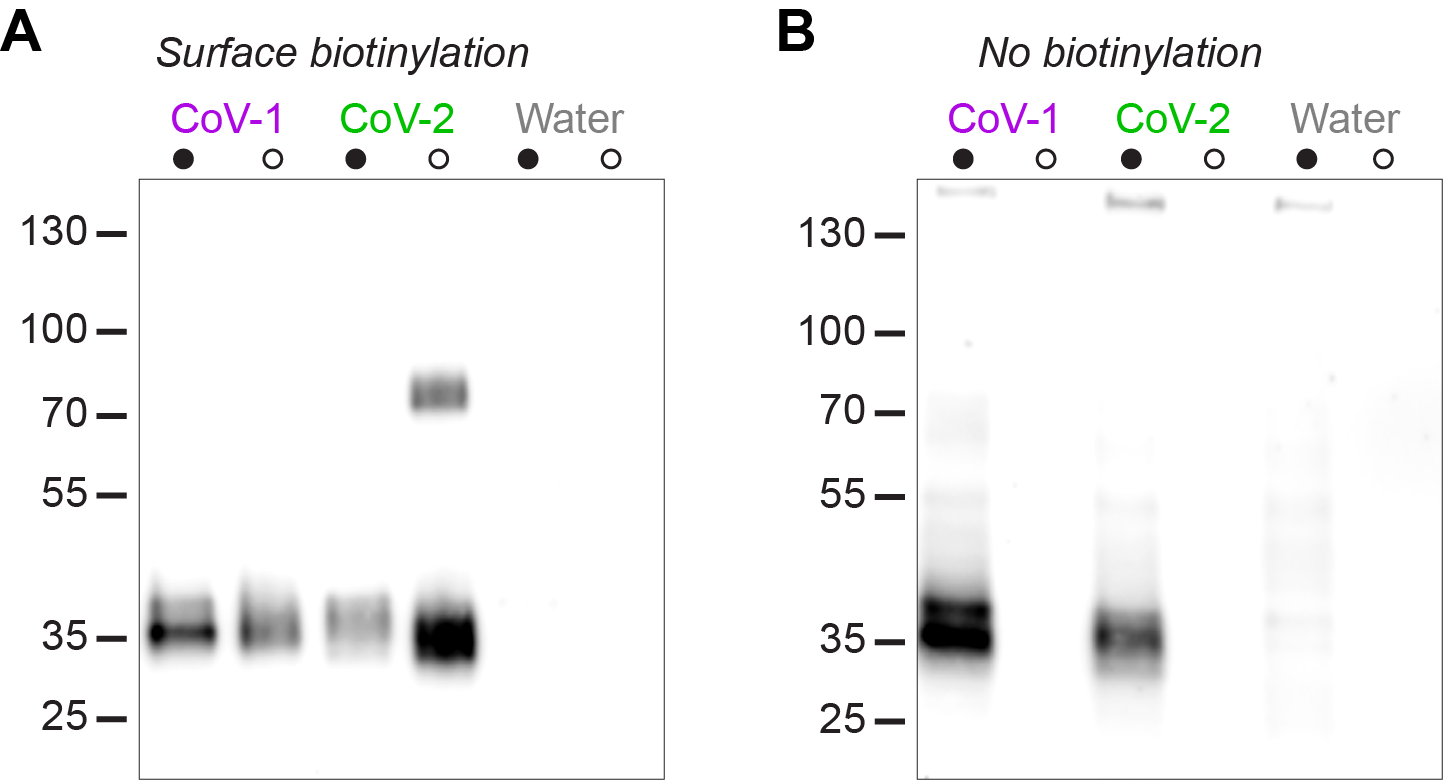

Supplement: Figure 2—figure supplement 3—source data 1. [file elife-84477-fig2-figsupp3-data1.zip › Figure 2-Figure Suppement 3-source data 1/Summary_Orf3a_oocytes_surface_biotinylation_experiment.png]

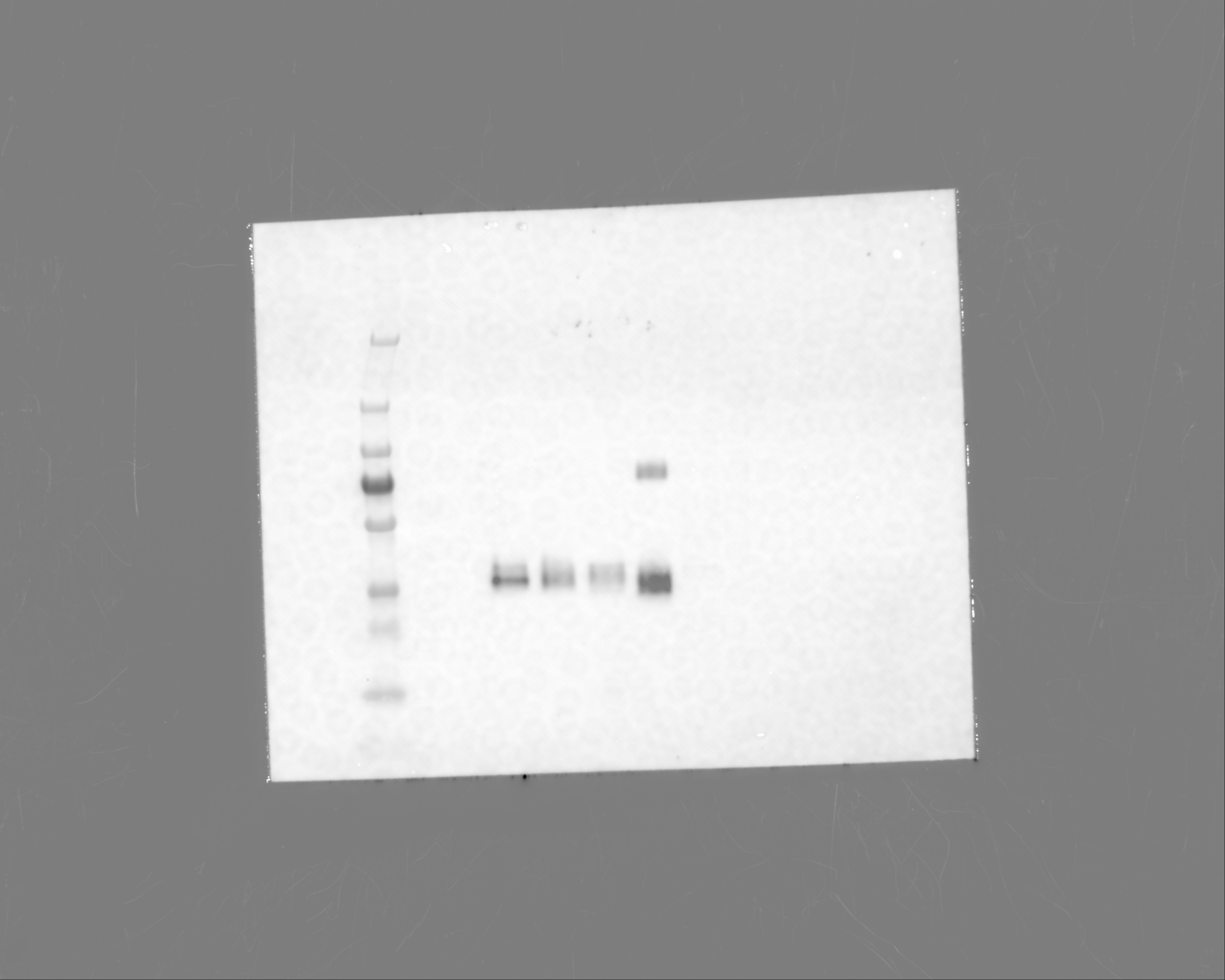

Supplement: Figure 2—figure supplement 3—source data 1. [file elife-84477-fig2-figsupp3-data1.zip › Figure 2-Figure Suppement 3-source data 1/Orf3a_oocyte_biotinylation samples_standardsmerged.tif]

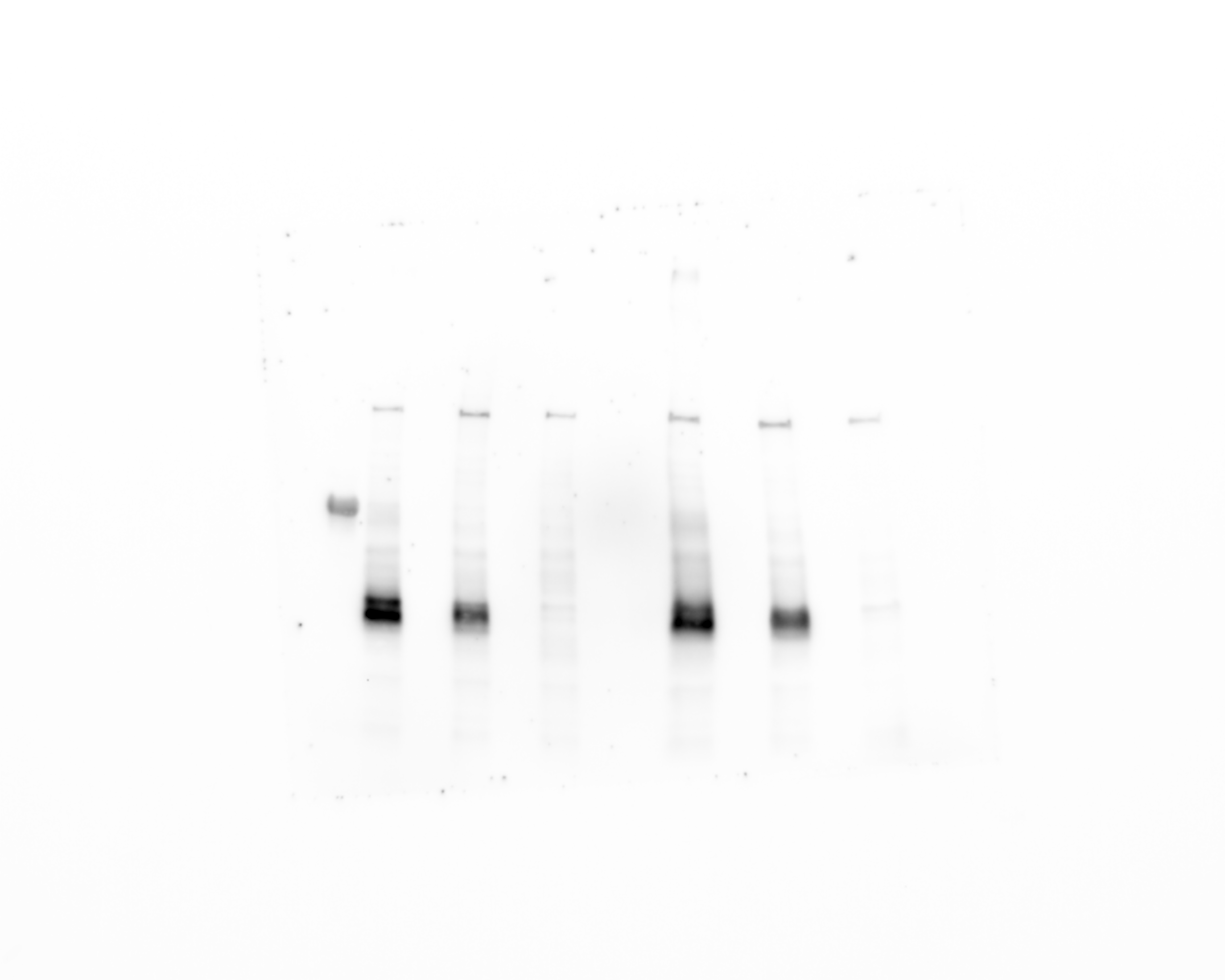

Supplement: Figure 2—figure supplement 3—source data 1. [file elife-84477-fig2-figsupp3-data1.zip › Figure 2-Figure Suppement 3-source data 1/Orf3a_oocyte_no biotinylation reagent_blot_alone.tif]

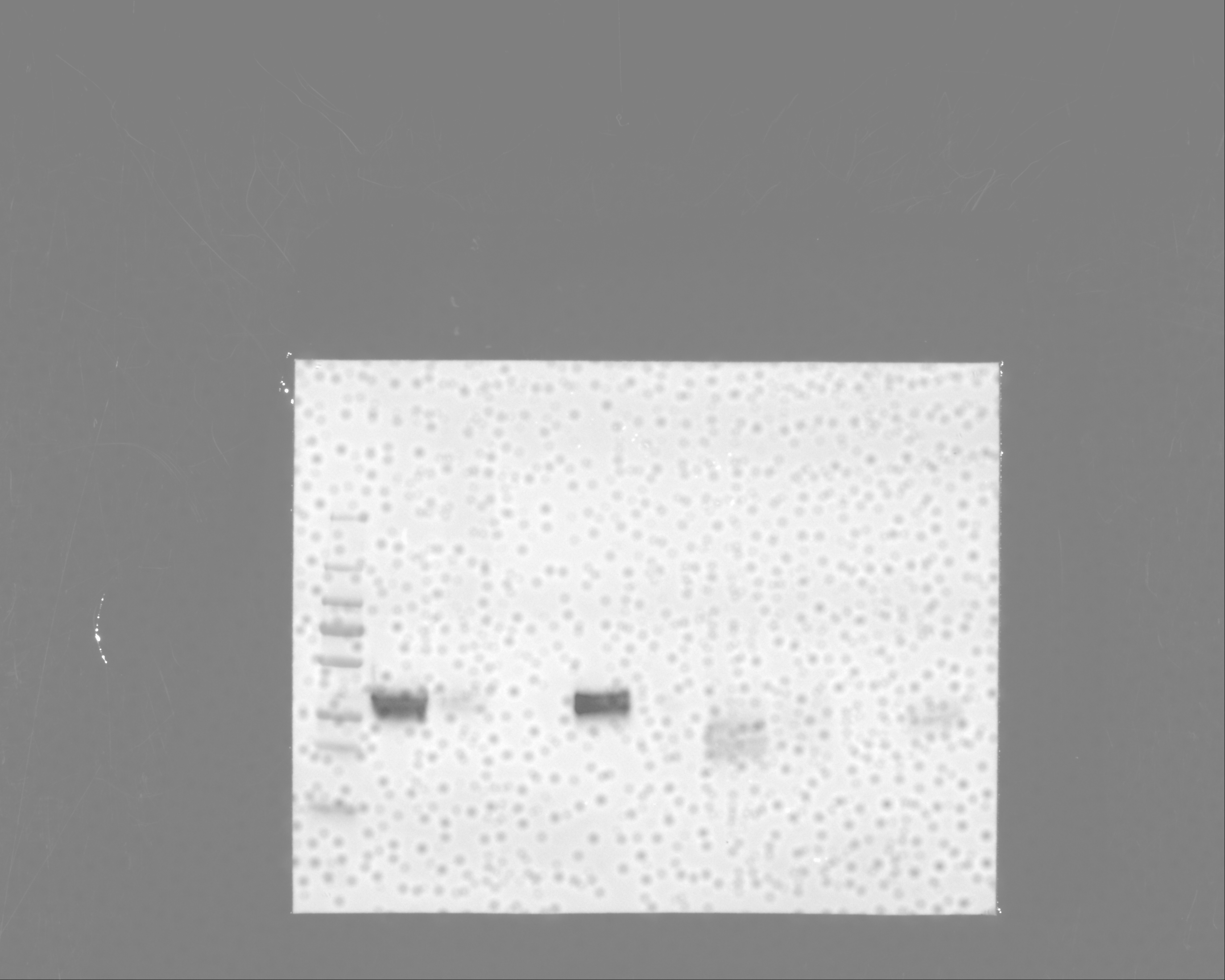

Supplement: Figure 6—source data 1. [file elife-84477-fig6-data1.zip › Figure 6-source data 1/SARS_CoV2_Orf3a_50ug_pulldown_first4lanes_strep_blot_merged.tif]

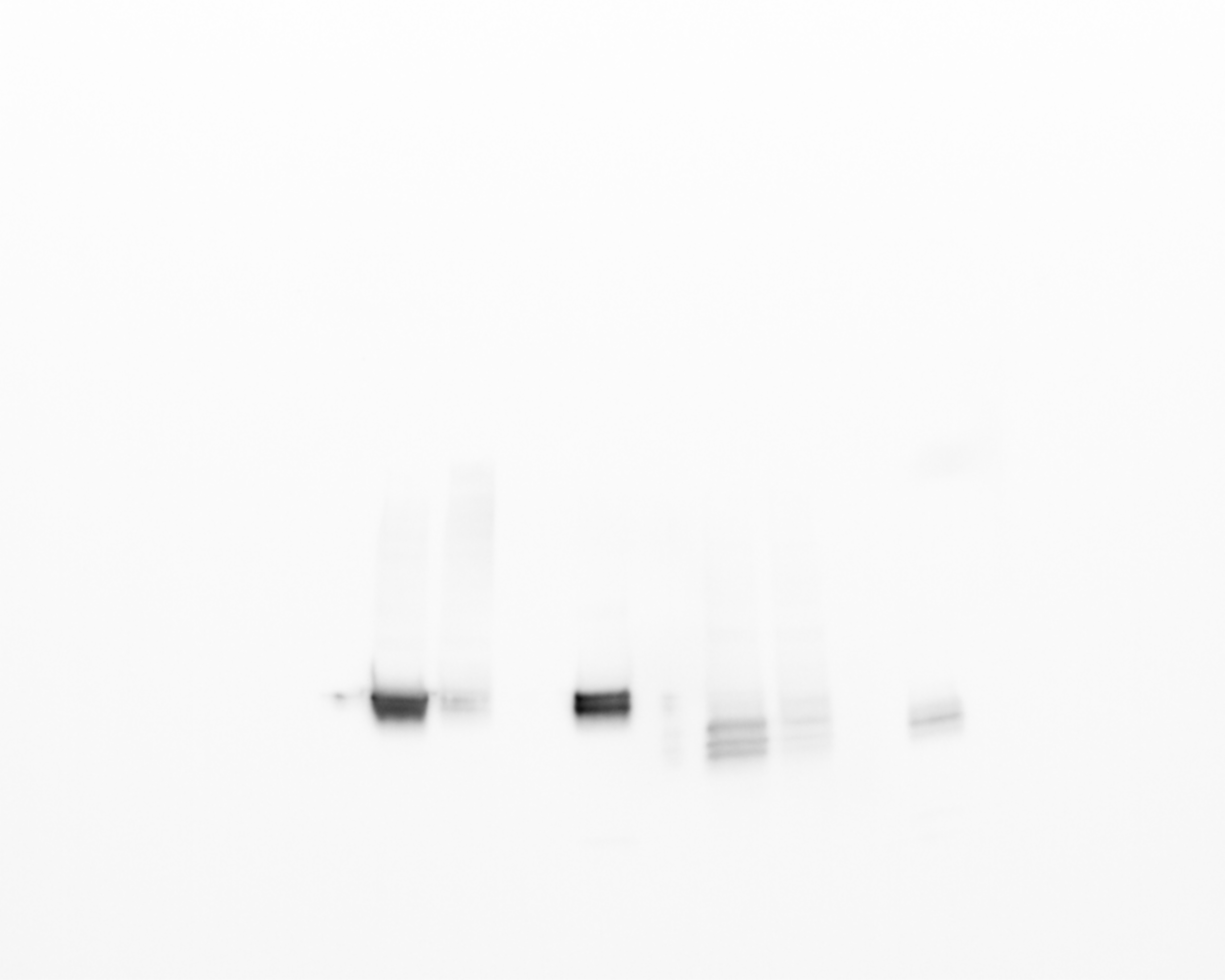

Supplement: Figure 6—source data 1. [file elife-84477-fig6-data1.zip › Figure 6-source data 1/SARS_CoV2_Orf3a_50ug_pulldown_first4lanes_strep_blot_only.tif]

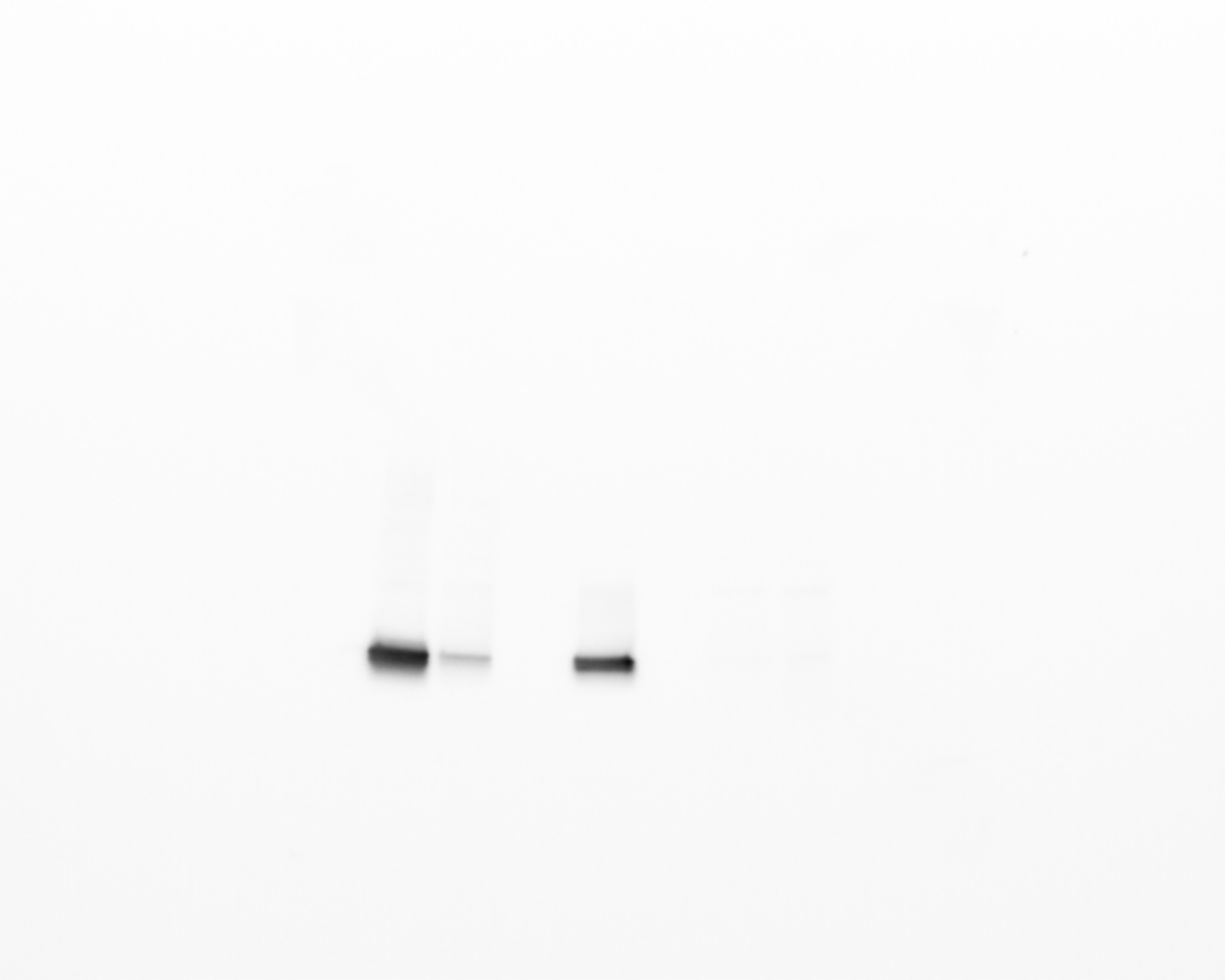

Supplement: Figure 6—source data 1. [file elife-84477-fig6-data1.zip › Figure 6-source data 1/SARS_CoV_1_Orf3a_50ug_and_no_protein_control_strep_blot_alone_a.tif]

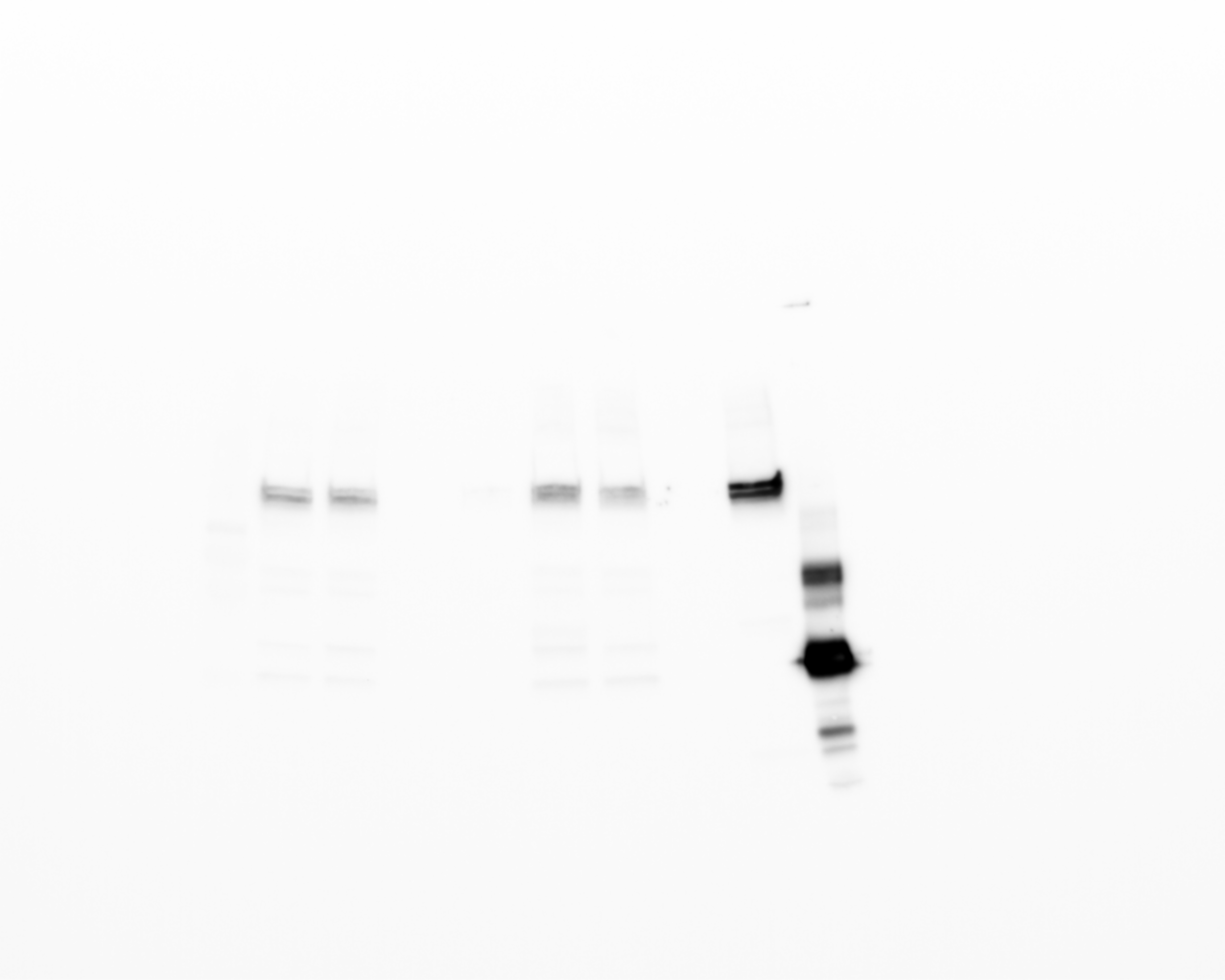

Supplement: Figure 6—source data 1. [file elife-84477-fig6-data1.zip › Figure 6-source data 1/SARS_CoV_1_CoV_2_Orf3a_50ug_pulldown_GFP_blot_only.tif]

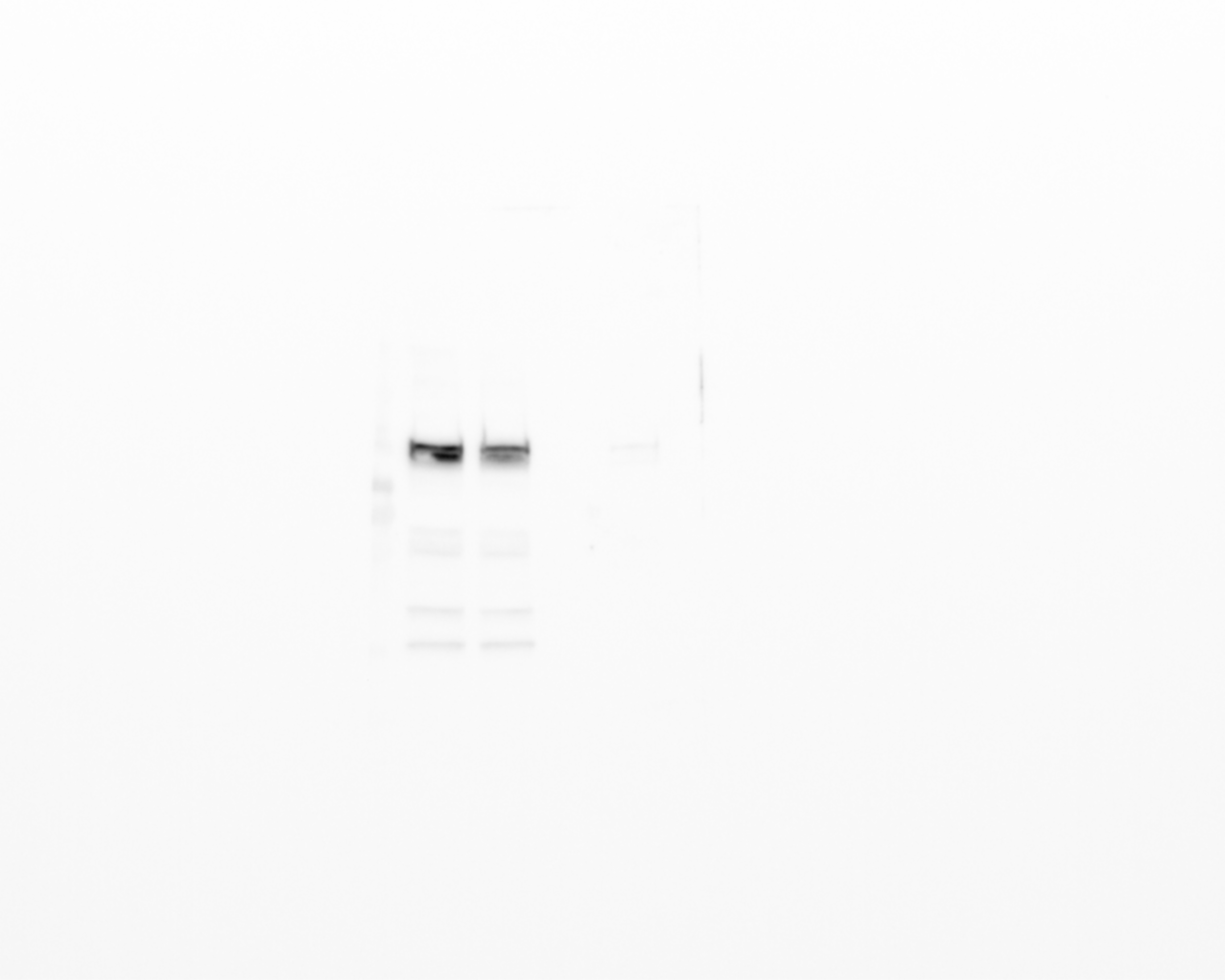

Supplement: Figure 6—source data 1. [file elife-84477-fig6-data1.zip › Figure 6-source data 1/No_protein_GFP_blot_only.tif]

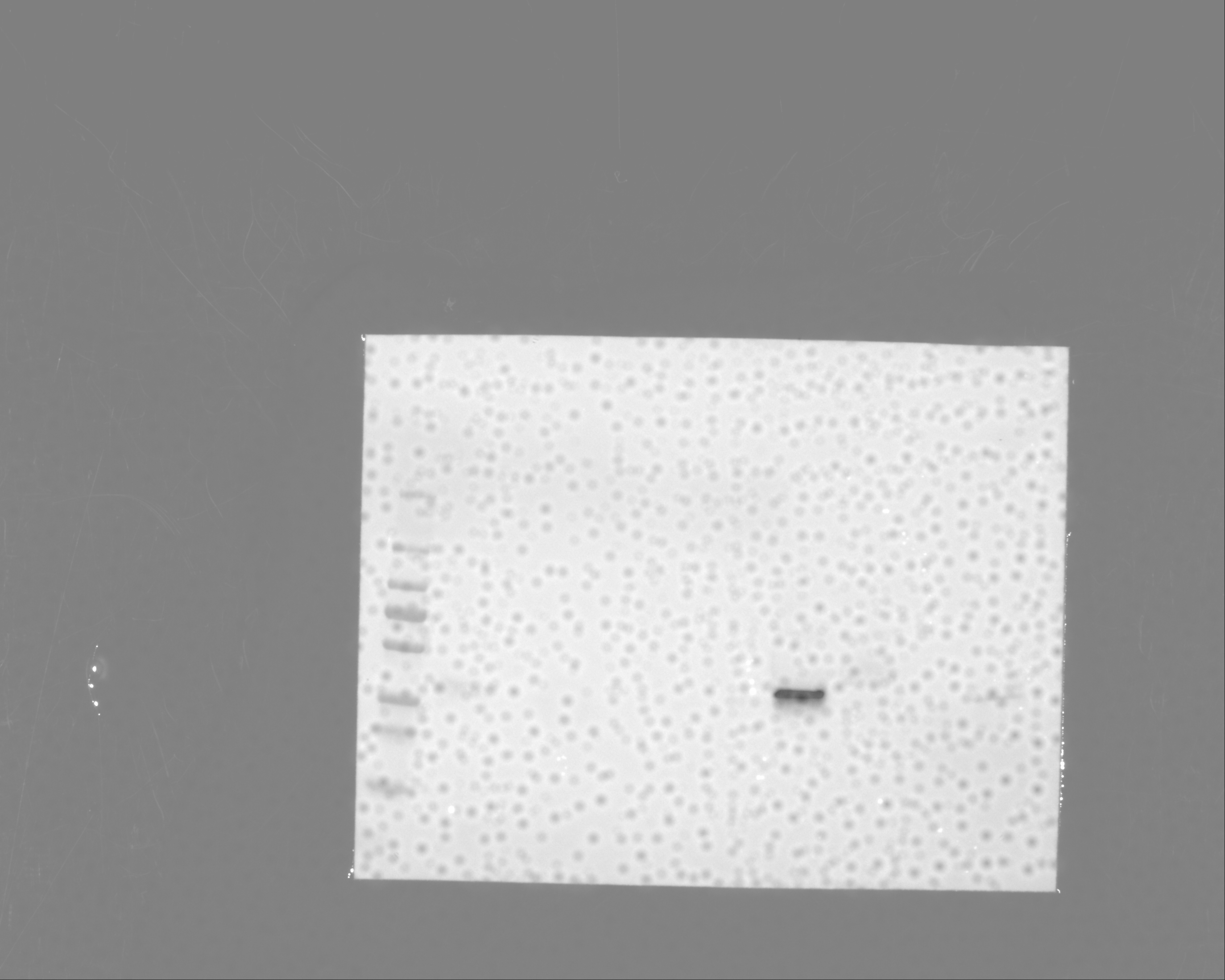

Supplement: Figure 6—source data 1. [file elife-84477-fig6-data1.zip › Figure 6-source data 1/SARS_CoV_2_Orf3a_05_5ug_pulldown_strep_blot_standardsmerged.tif]

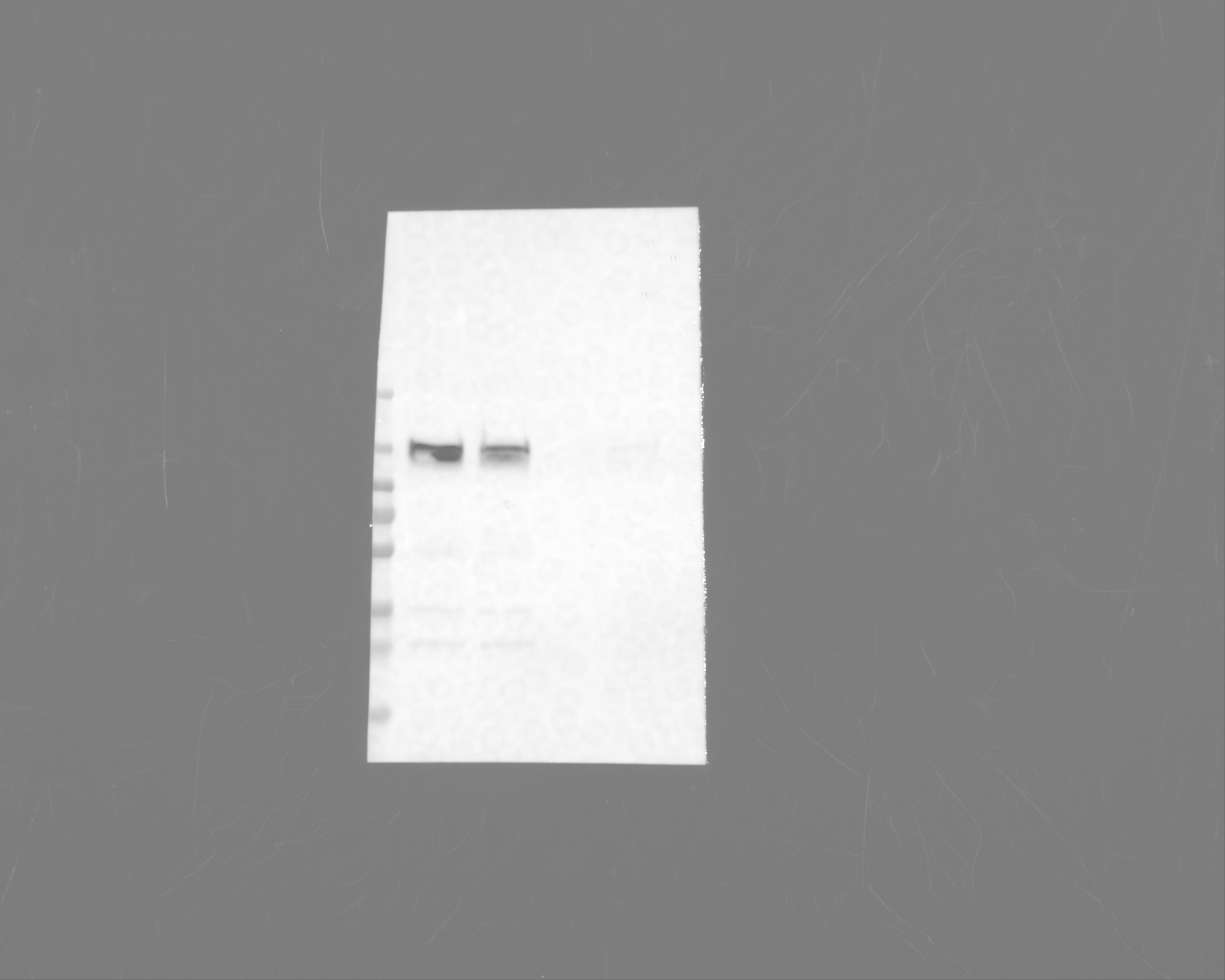

Supplement: Figure 6—source data 1. [file elife-84477-fig6-data1.zip › Figure 6-source data 1/No_protein_GFP_blot_stndardsmerged.tif]

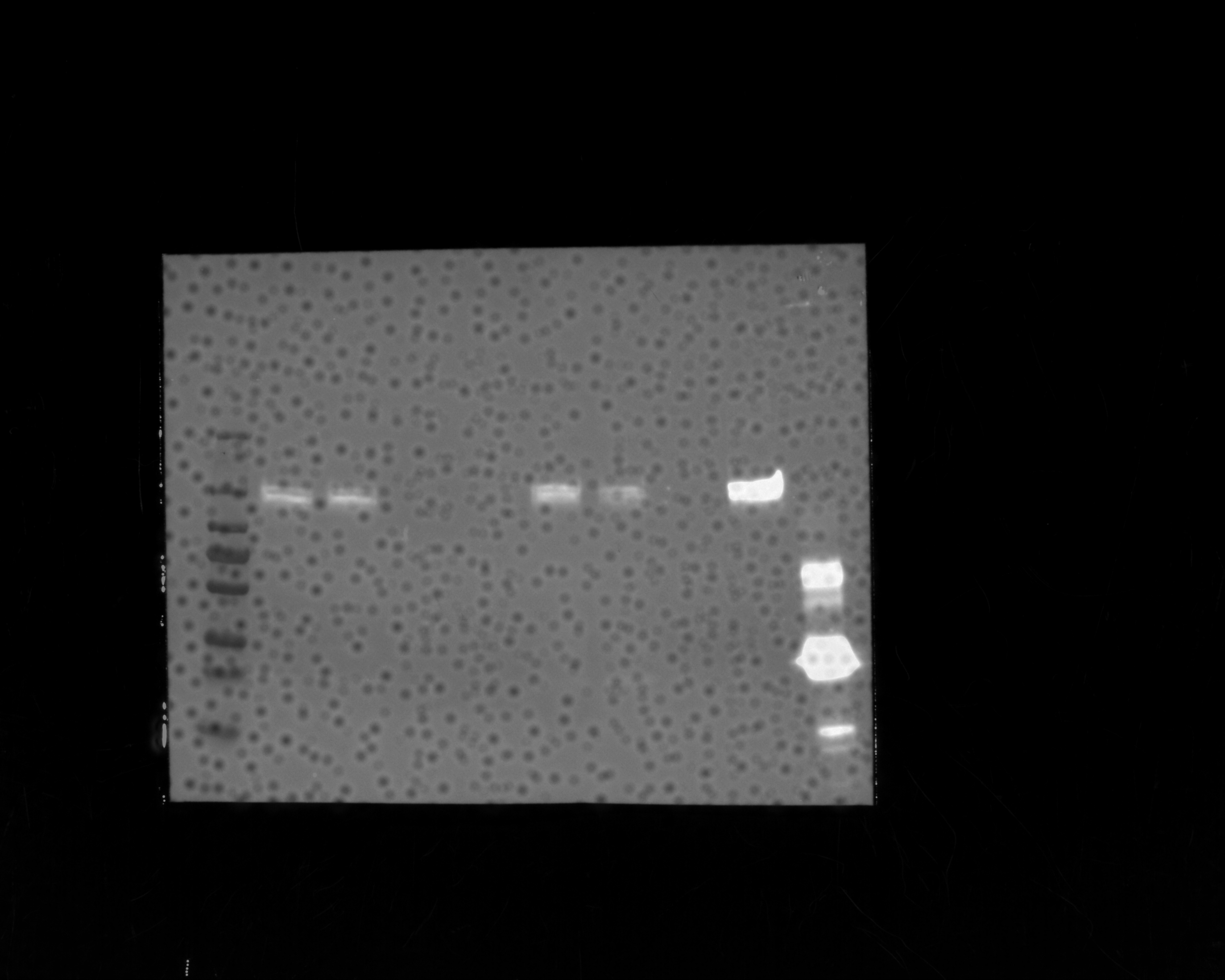

Supplement: Figure 6—source data 1. [file elife-84477-fig6-data1.zip › Figure 6-source data 1/SARS_CoV_1_CoV_2_Orf3a_50ug_pulldown_GFP_blot_merged.tif]

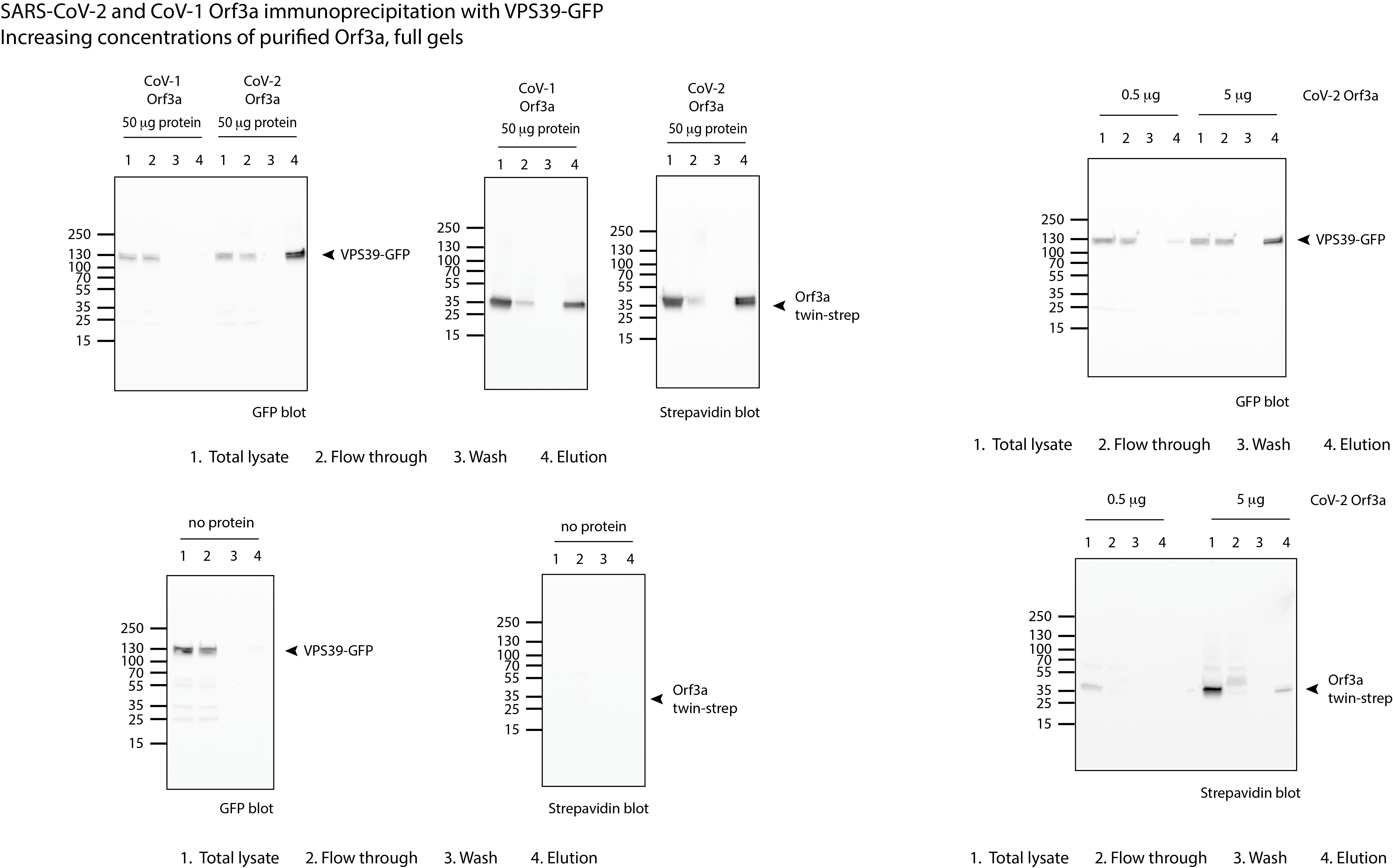

Supplement: Figure 6—source data 1. [file elife-84477-fig6-data1.zip › Figure 6-source data 1/Summary_immunoprecipitation_Orf3a_VPS39_fullgel.png]

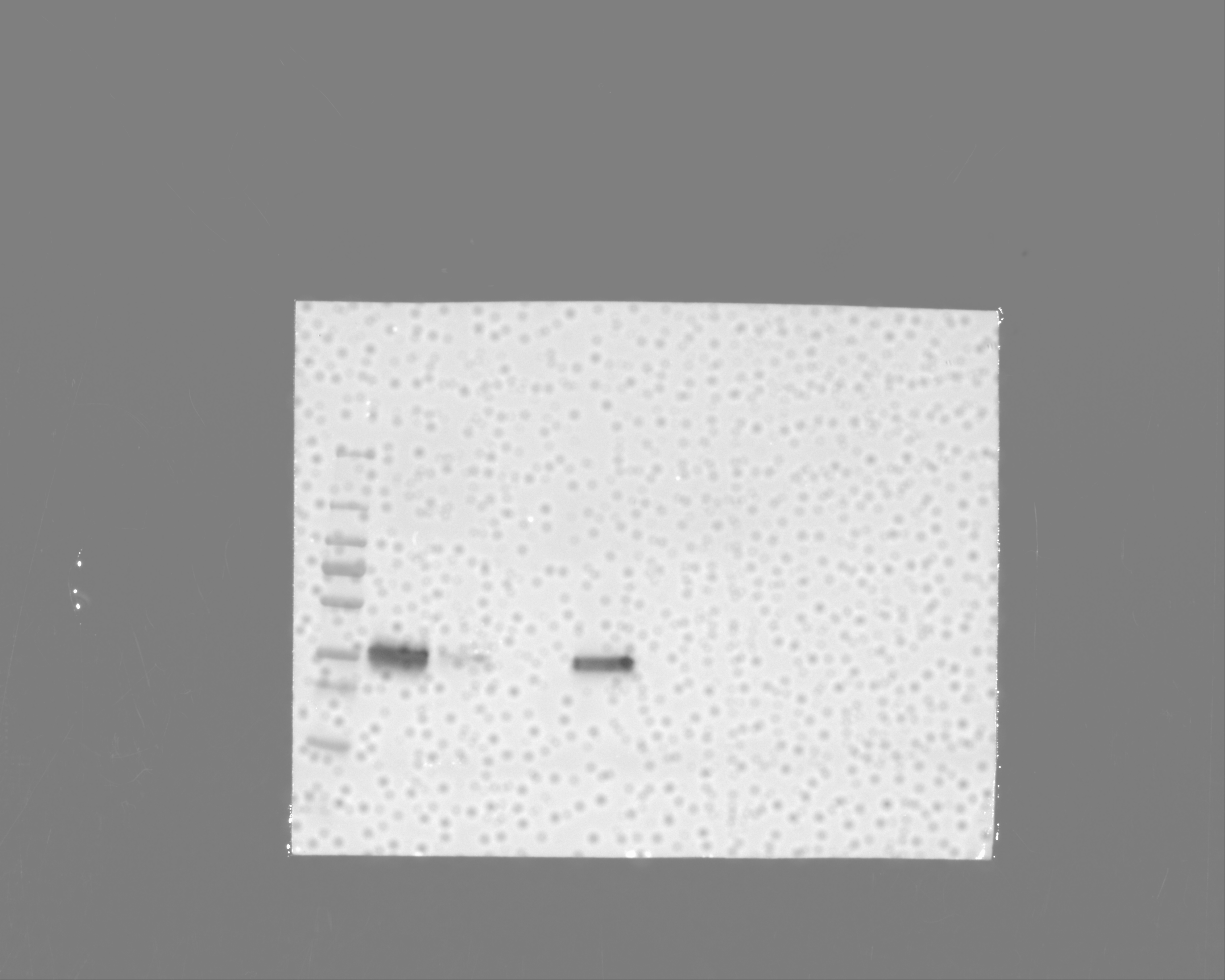

Supplement: Figure 6—source data 1. [file elife-84477-fig6-data1.zip › Figure 6-source data 1/SARS_CoV_1_Orf3a_50ug_and_no_protein_control_strep_blot_standardsmerged.tif]

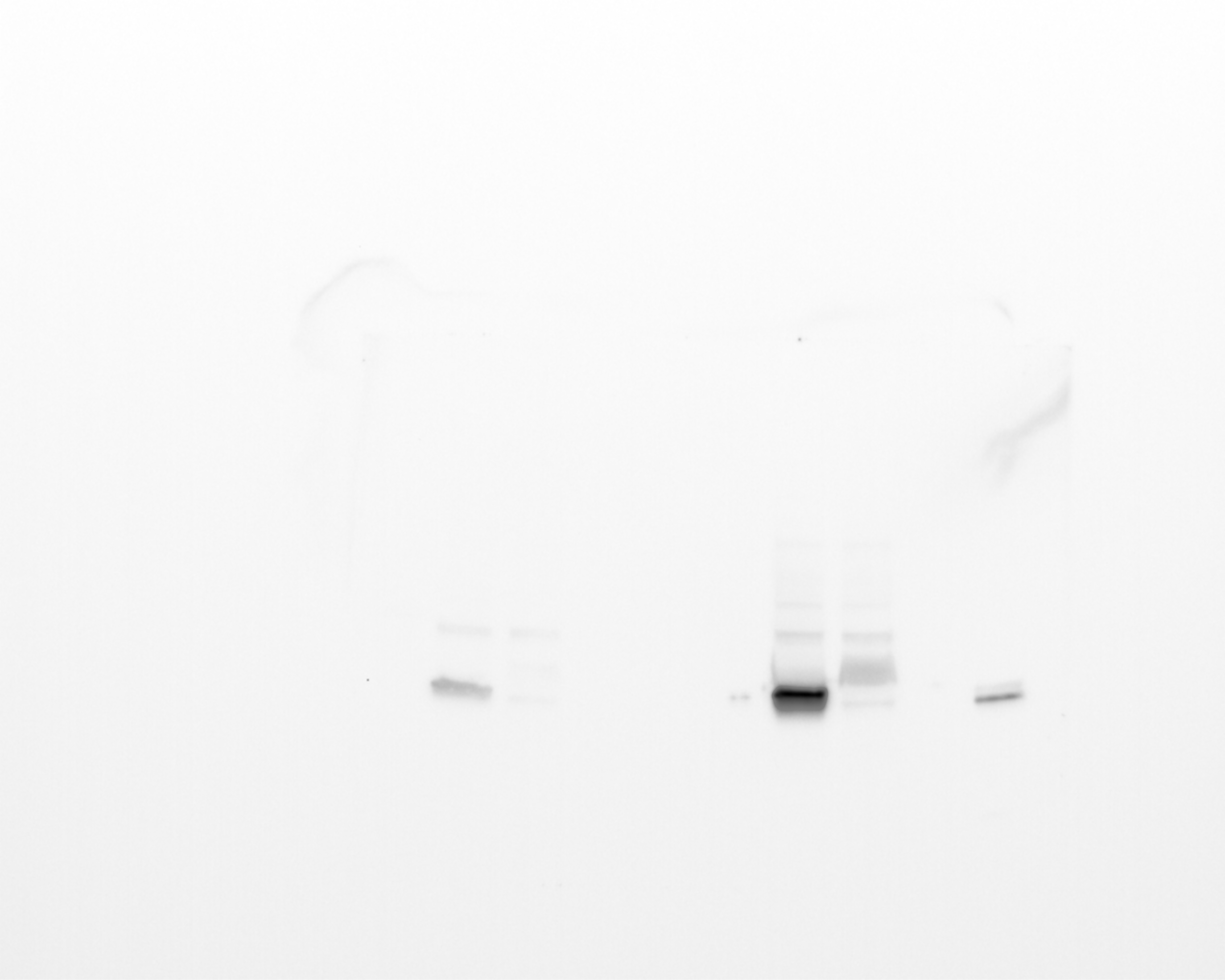

Supplement: Figure 6—source data 1. [file elife-84477-fig6-data1.zip › Figure 6-source data 1/SARS_CoV_2_Orf3a_05_5ug_pulldown_strep_blot_only.tif]

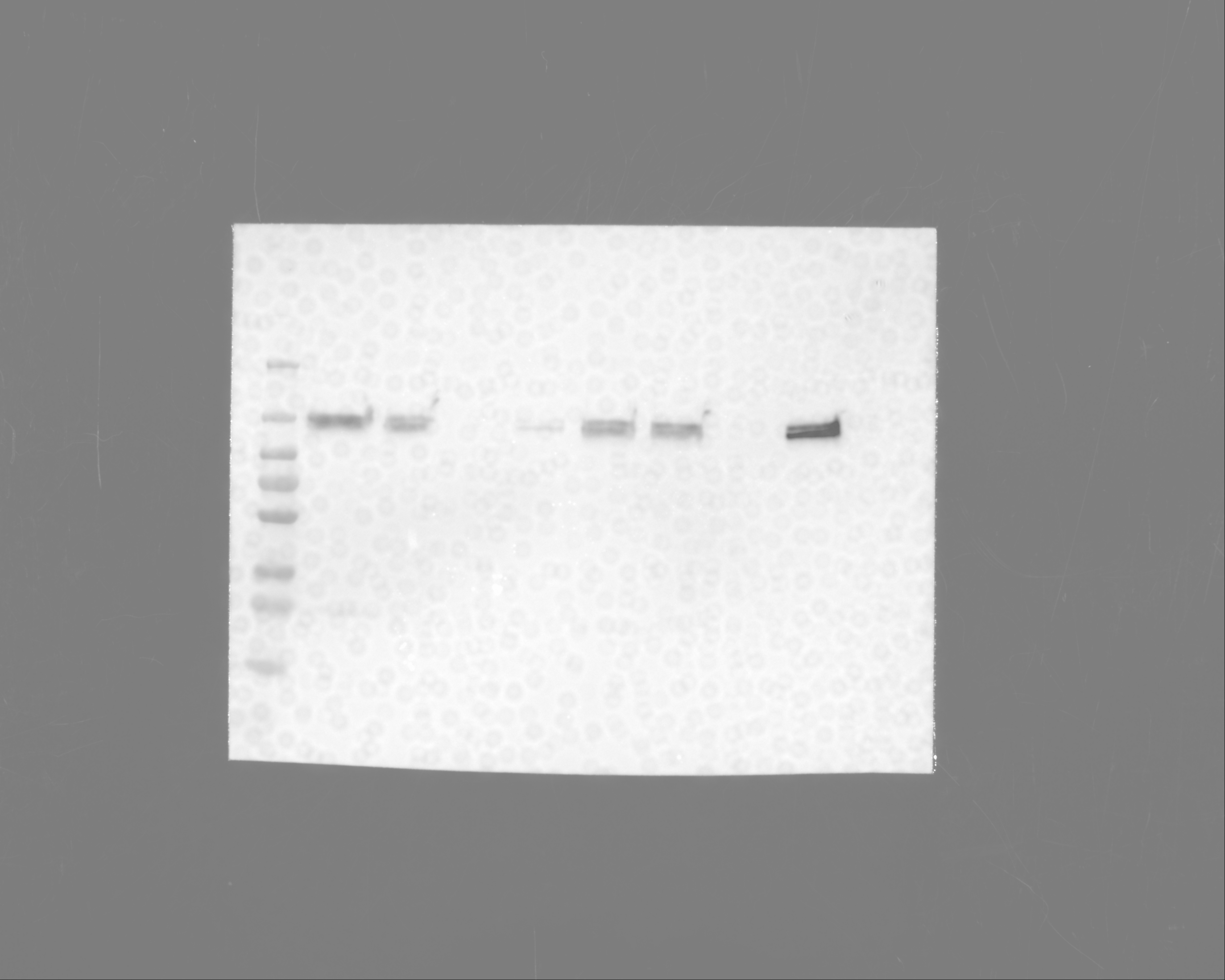

Supplement: Figure 6—source data 1. [file elife-84477-fig6-data1.zip › Figure 6-source data 1/SARS_CoV_2_Orf3a_05_5ug_pulldown_GFP_blot_standardsmerged.tif]

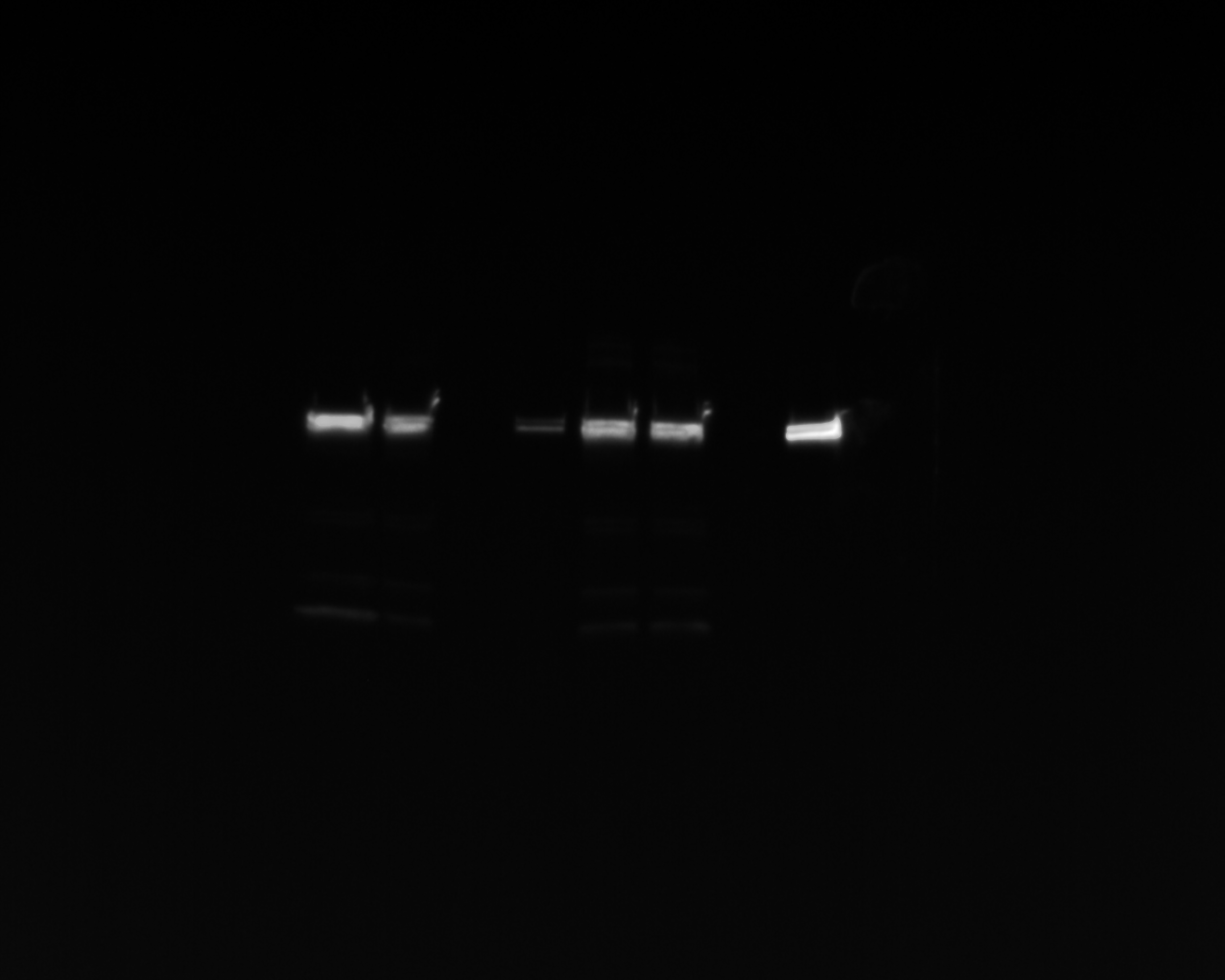

Supplement: Figure 6—source data 1. [file elife-84477-fig6-data1.zip › Figure 6-source data 1/SARS_CoV_2_Orf3a_05_5ug_pulldown_GFP_blot_only.tif]

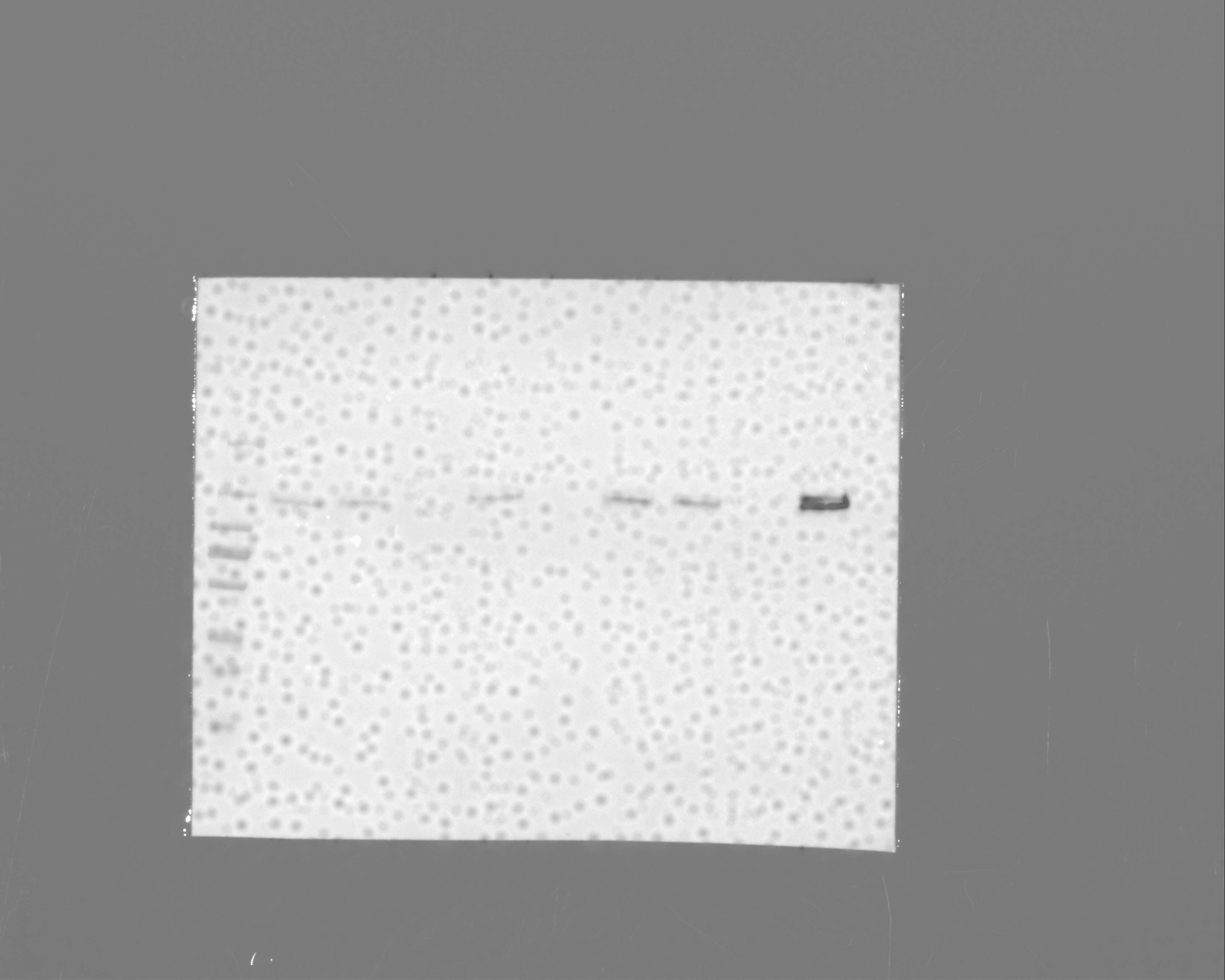

Supplement: Figure 6—source data 2. [file elife-84477-fig6-data2.zip › Figure 6-source data 2/SARS_CoV_1_Orf3a_wt_and_LC_mut_pulldown_50ug_GFP_blot_standardsmerged.tif]

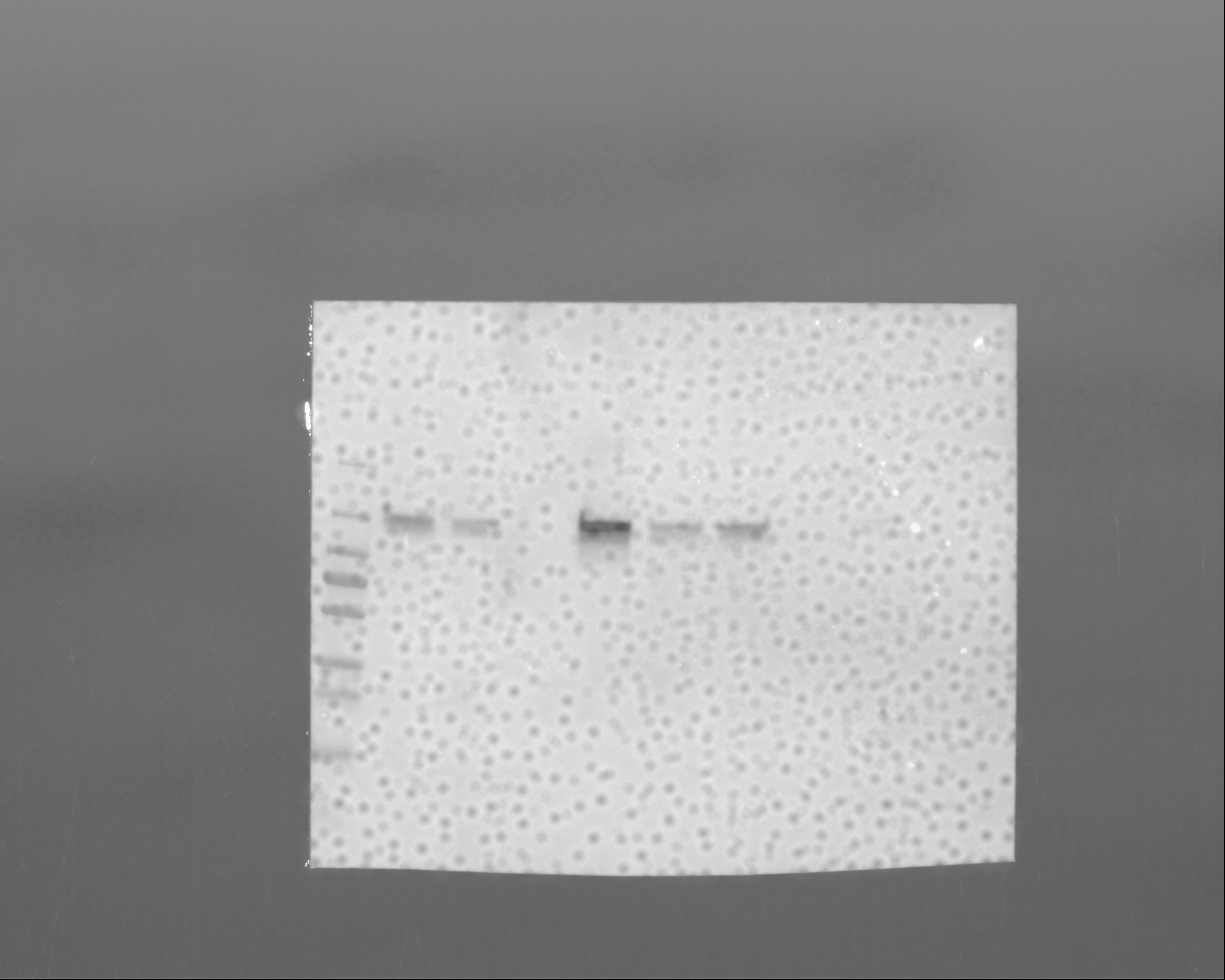

Supplement: Figure 6—source data 2. [file elife-84477-fig6-data2.zip › Figure 6-source data 2/SARS_CoV_2_Orf3a_wt_and_LC_mut_pulldown_5ug_GFP_blot_standardsmerged.tif]

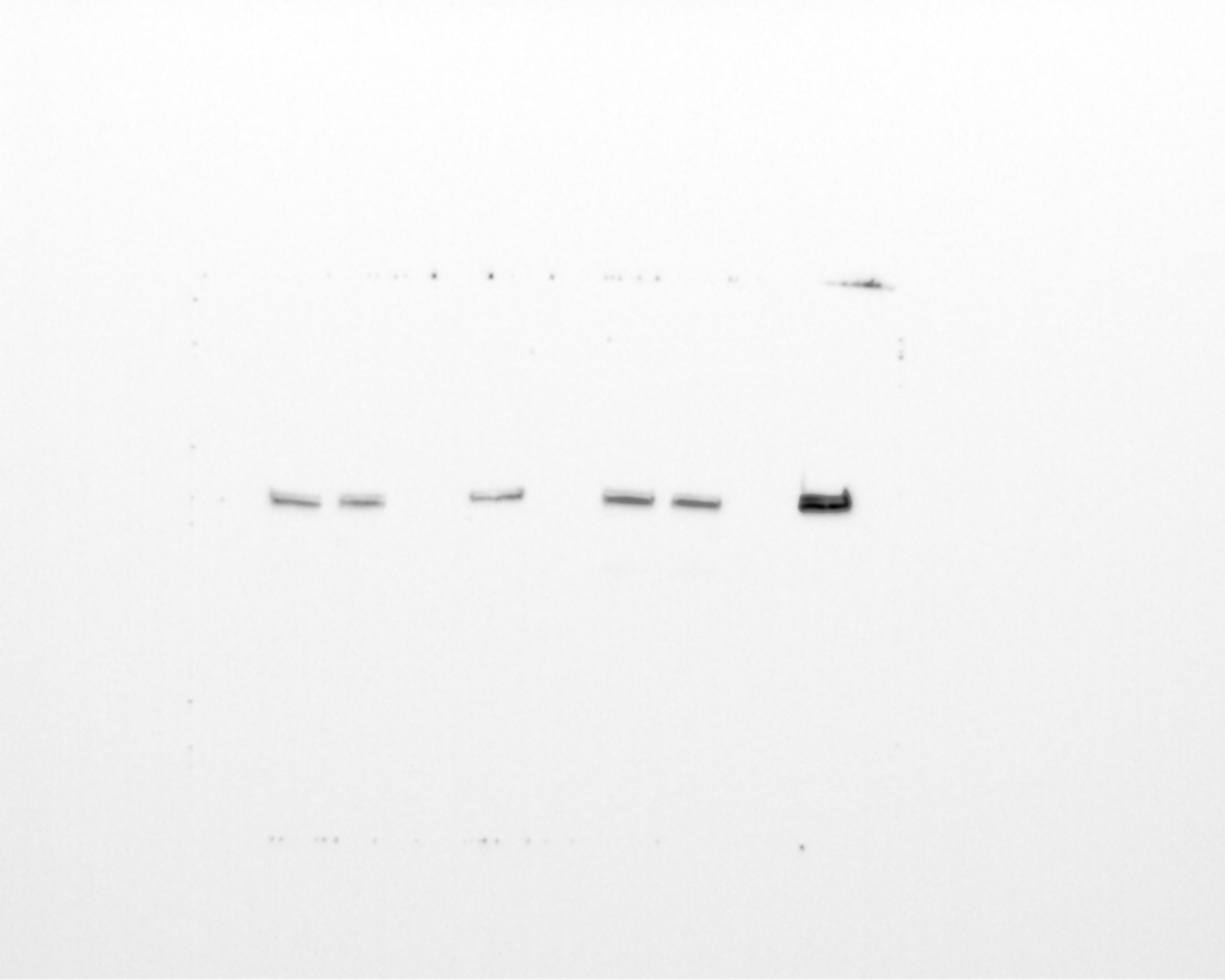

Supplement: Figure 6—source data 2. [file elife-84477-fig6-data2.zip › Figure 6-source data 2/SARS_CoV_1_Orf3a_wt_and_LC_mut_pulldown_50ug_GFP_blot_only.tif]

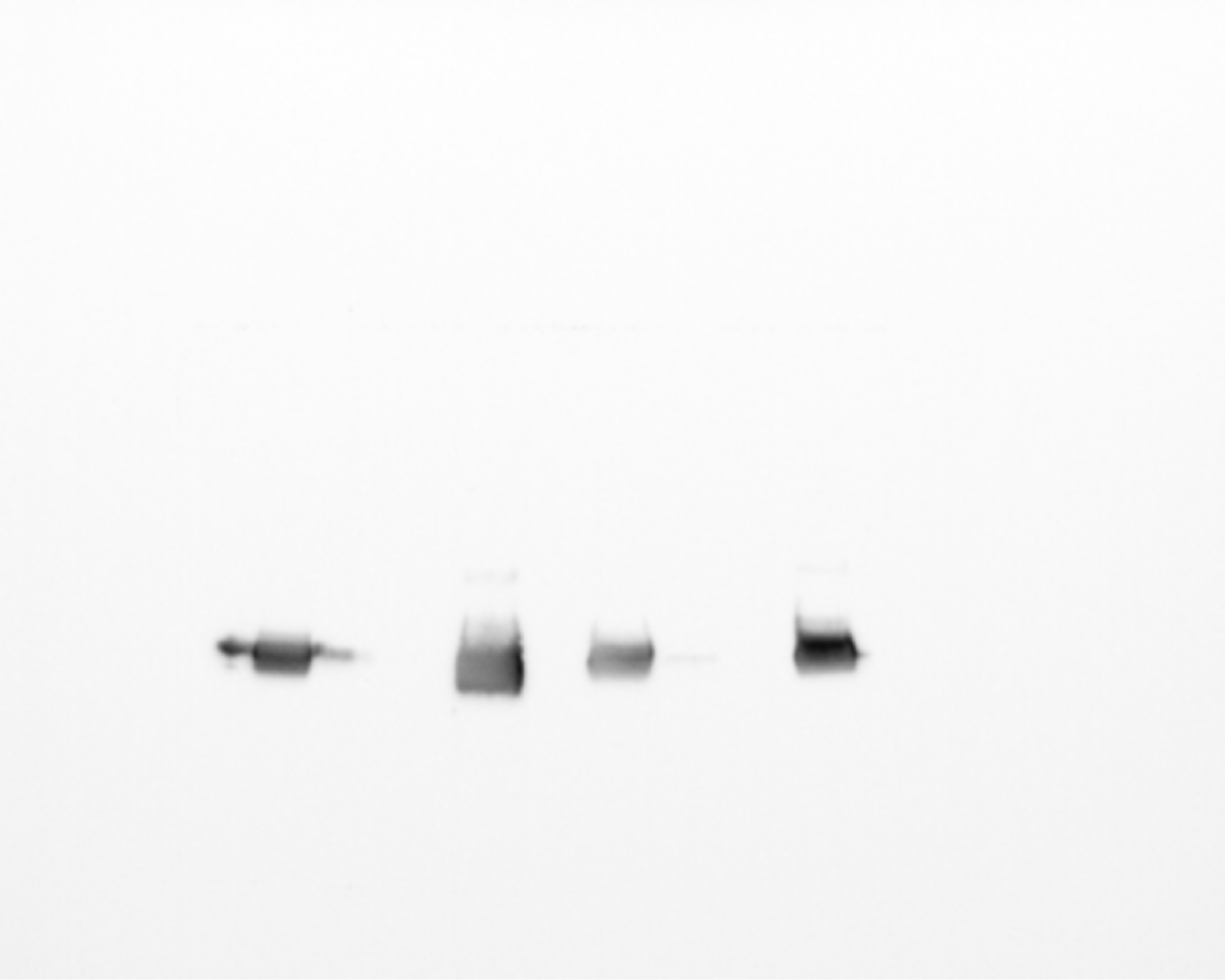

Supplement: Figure 6—source data 2. [file elife-84477-fig6-data2.zip › Figure 6-source data 2/SARS_CoV_1_Orf3a_wt_and_LC_mut_pulldown_50ug_strep_blot_only.tif]

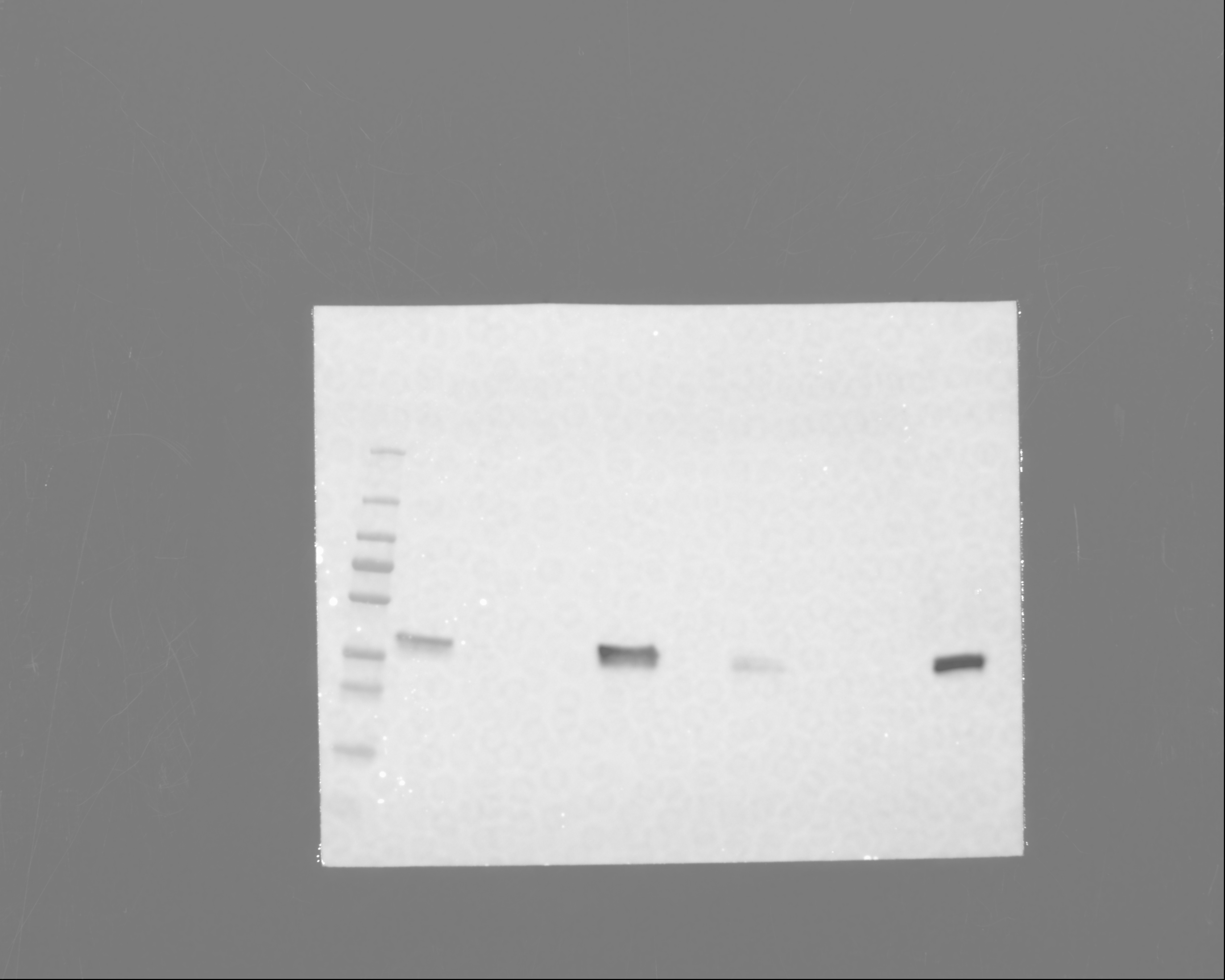

Supplement: Figure 6—source data 2. [file elife-84477-fig6-data2.zip › Figure 6-source data 2/SARS_CoV_2_Orf3a_wt_and_LC_mut_pulldown_5ug_strep_blot_standardsmerged.tif]

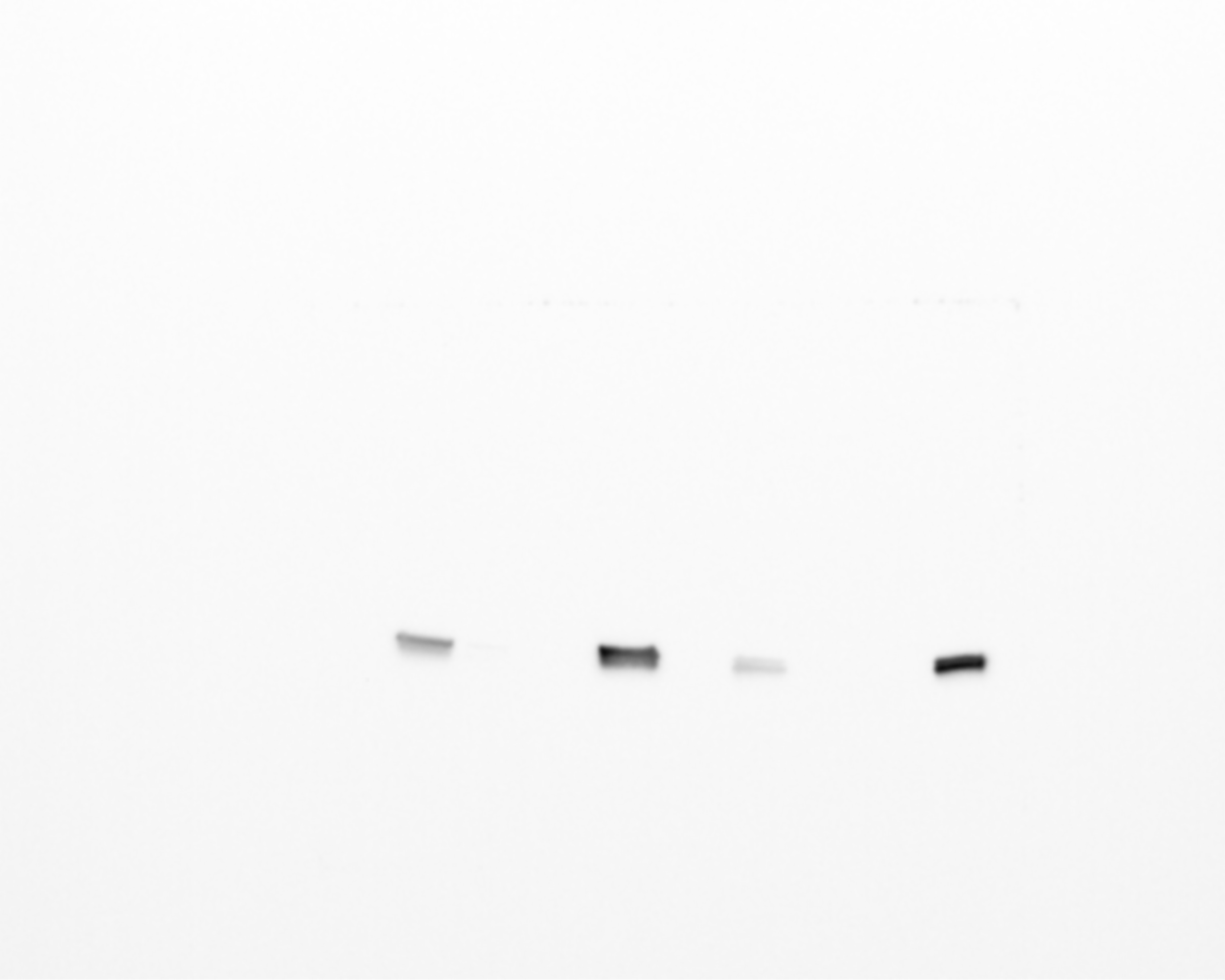

Supplement: Figure 6—source data 2. [file elife-84477-fig6-data2.zip › Figure 6-source data 2/SARS_CoV_2_Orf3a_wt_and_LC_mut_pulldown_5ug_strep_blot_only.tif]

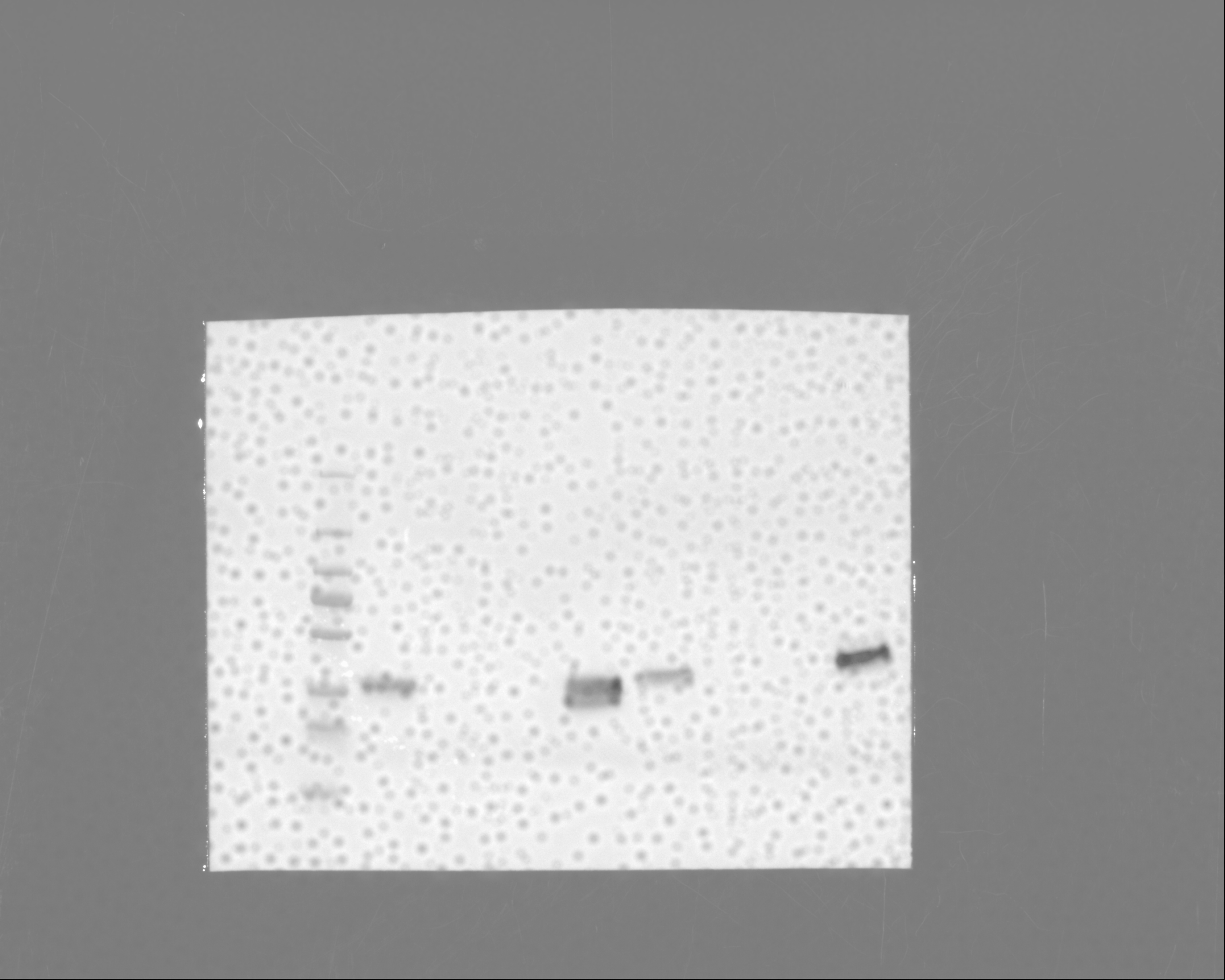

Supplement: Figure 6—source data 2. [file elife-84477-fig6-data2.zip › Figure 6-source data 2/SARS_CoV_1_Orf3a_wt_and_LC_mut_pulldown_5ug_strep_blot_standardsmerged.tif]

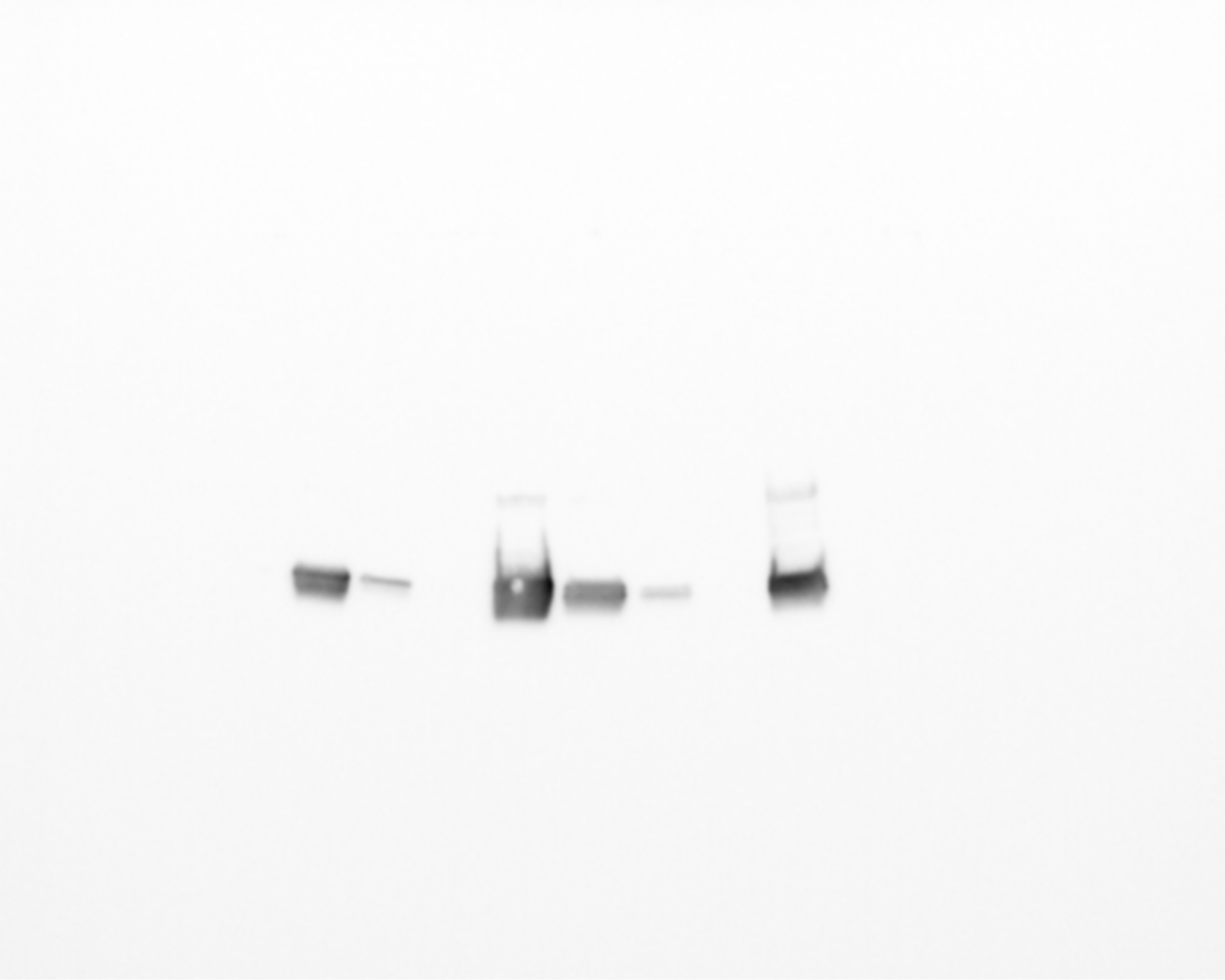

Supplement: Figure 6—source data 2. [file elife-84477-fig6-data2.zip › Figure 6-source data 2/SARS_CoV_2_Orf3a_wt_and_LC_mut_pulldown_50ug_strep_blot_only.tif]

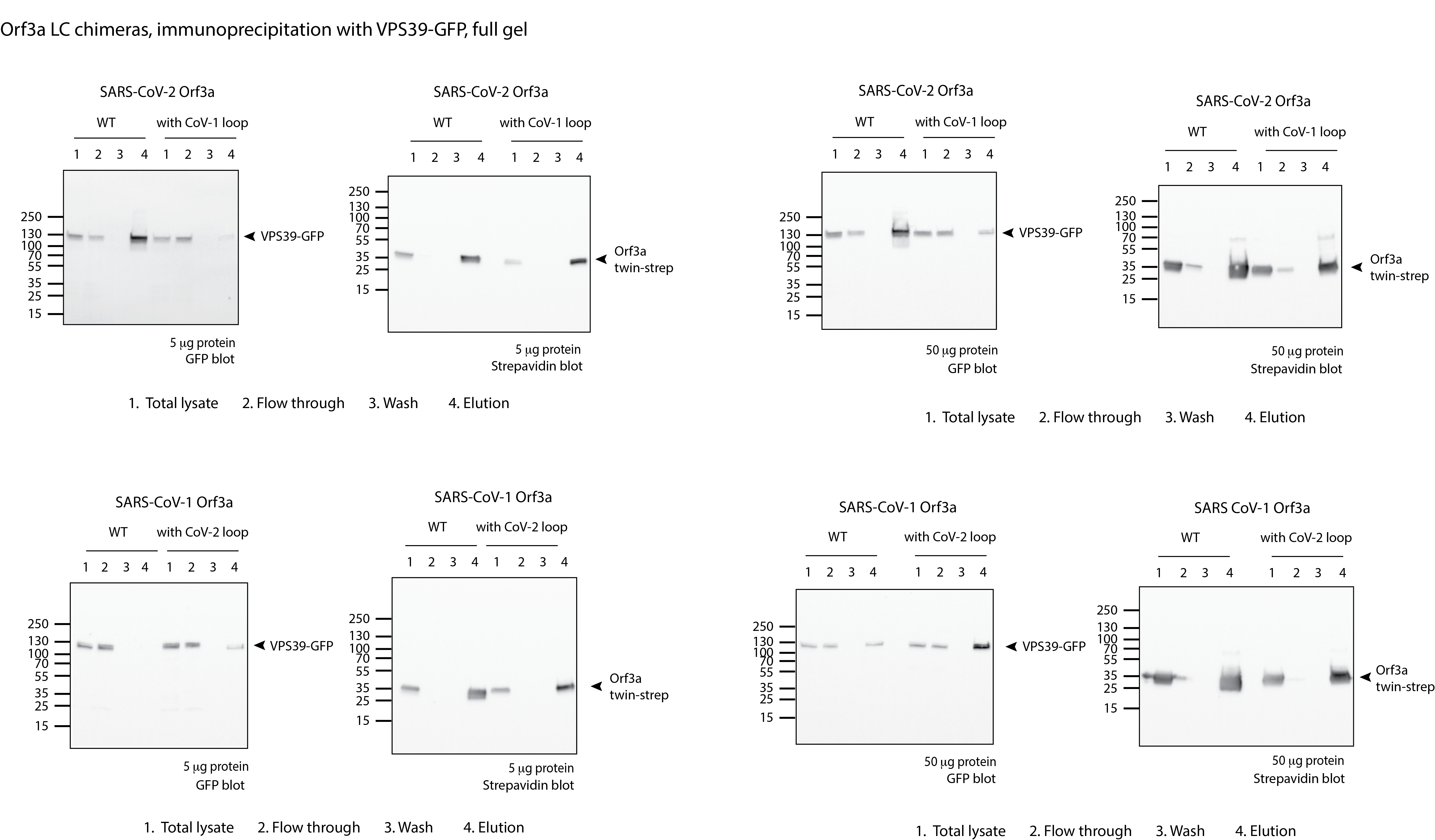

Supplement: Figure 6—source data 2. [file elife-84477-fig6-data2.zip › Figure 6-source data 2/Summary_immunoprecipitation_Orf3a_VPS39_LC_fullgel.png]

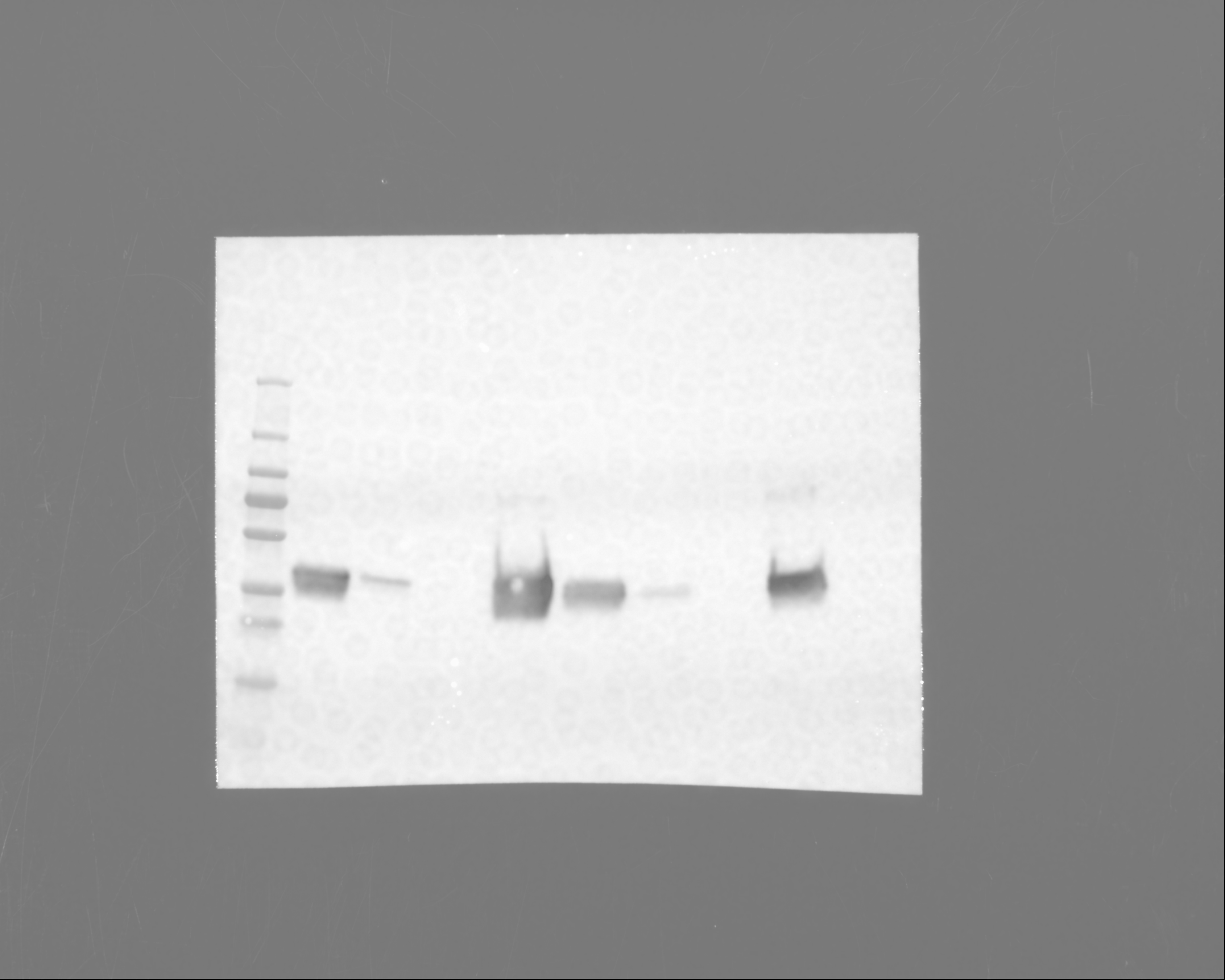

Supplement: Figure 6—source data 2. [file elife-84477-fig6-data2.zip › Figure 6-source data 2/SARS_CoV_2_Orf3a_wt_and_LC_mut_pulldown_50ug_strep_blot_standardsmerged.tif]

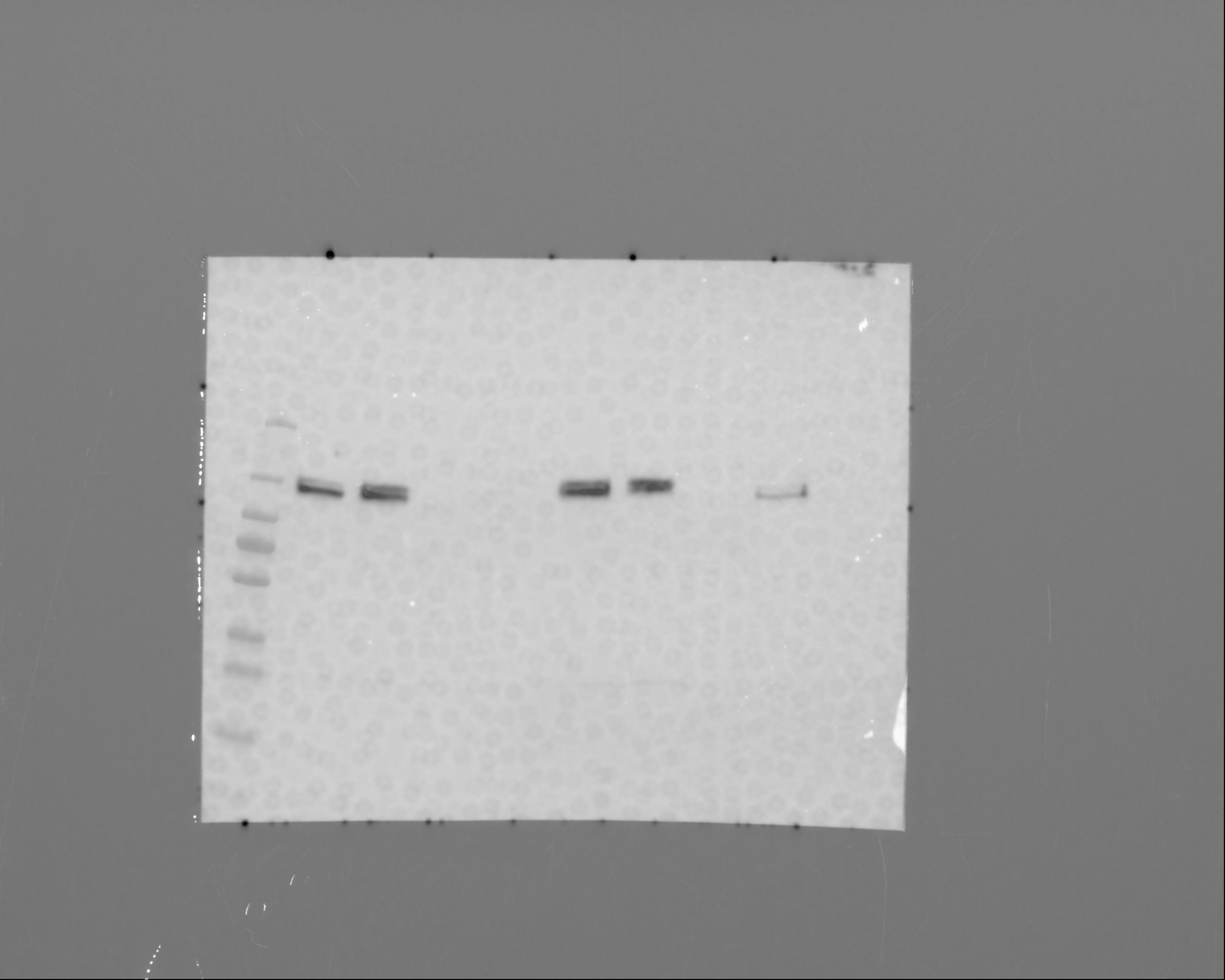

Supplement: Figure 6—source data 2. [file elife-84477-fig6-data2.zip › Figure 6-source data 2/SARS_CoV_1_Orf3a_wt_and_LC_mut_pulldown_5ug_GFP_blot_standardsmerged.tif]

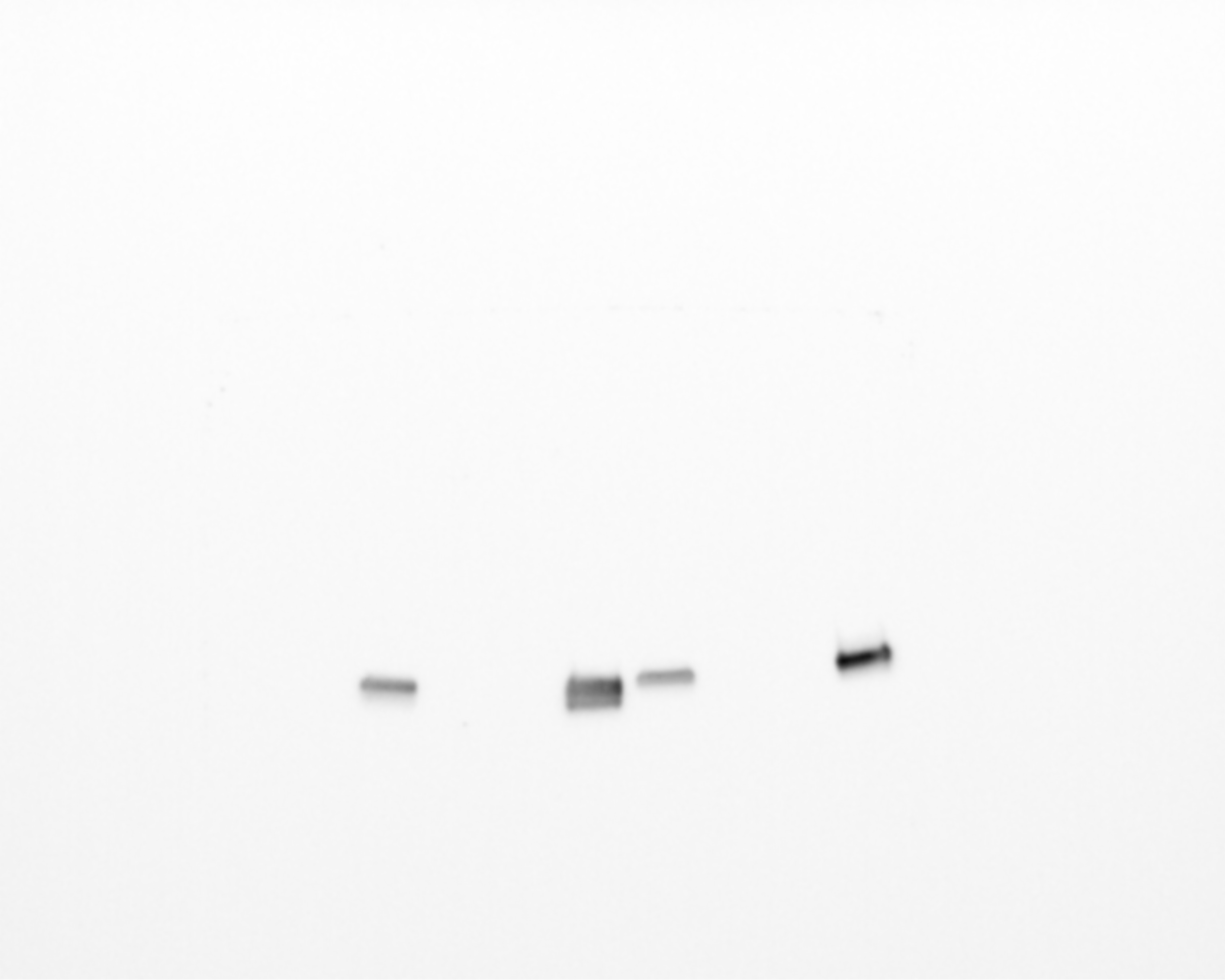

Supplement: Figure 6—source data 2. [file elife-84477-fig6-data2.zip › Figure 6-source data 2/SARS_CoV_1_Orf3a_wt_and_LC_mut_pulldown_5ug_strep_blot_only.tif]

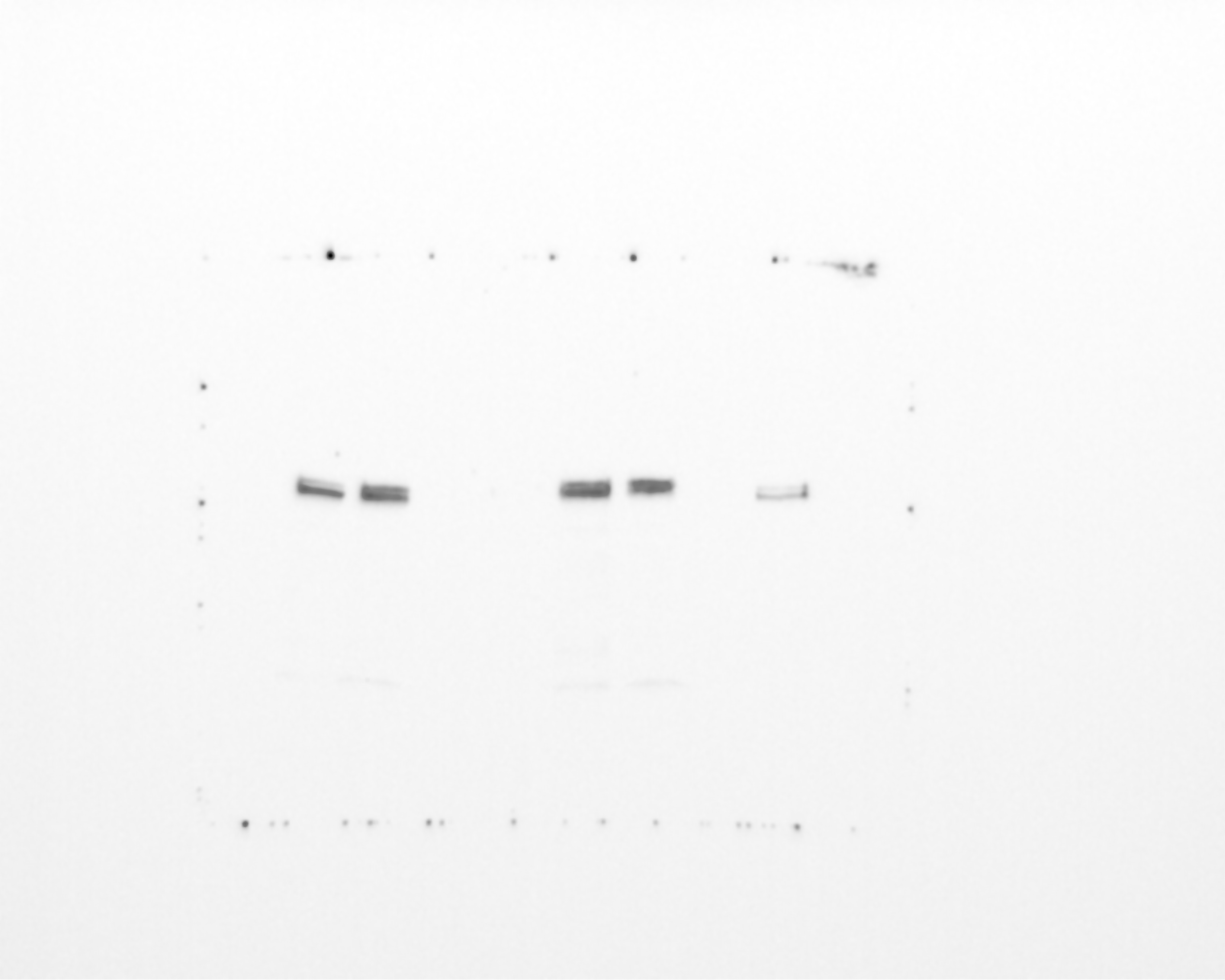

Supplement: Figure 6—source data 2. [file elife-84477-fig6-data2.zip › Figure 6-source data 2/SARS_CoV_1_Orf3a_wt_and_LC_mut_pulldown_5ug_GFP_blot_only.tif]

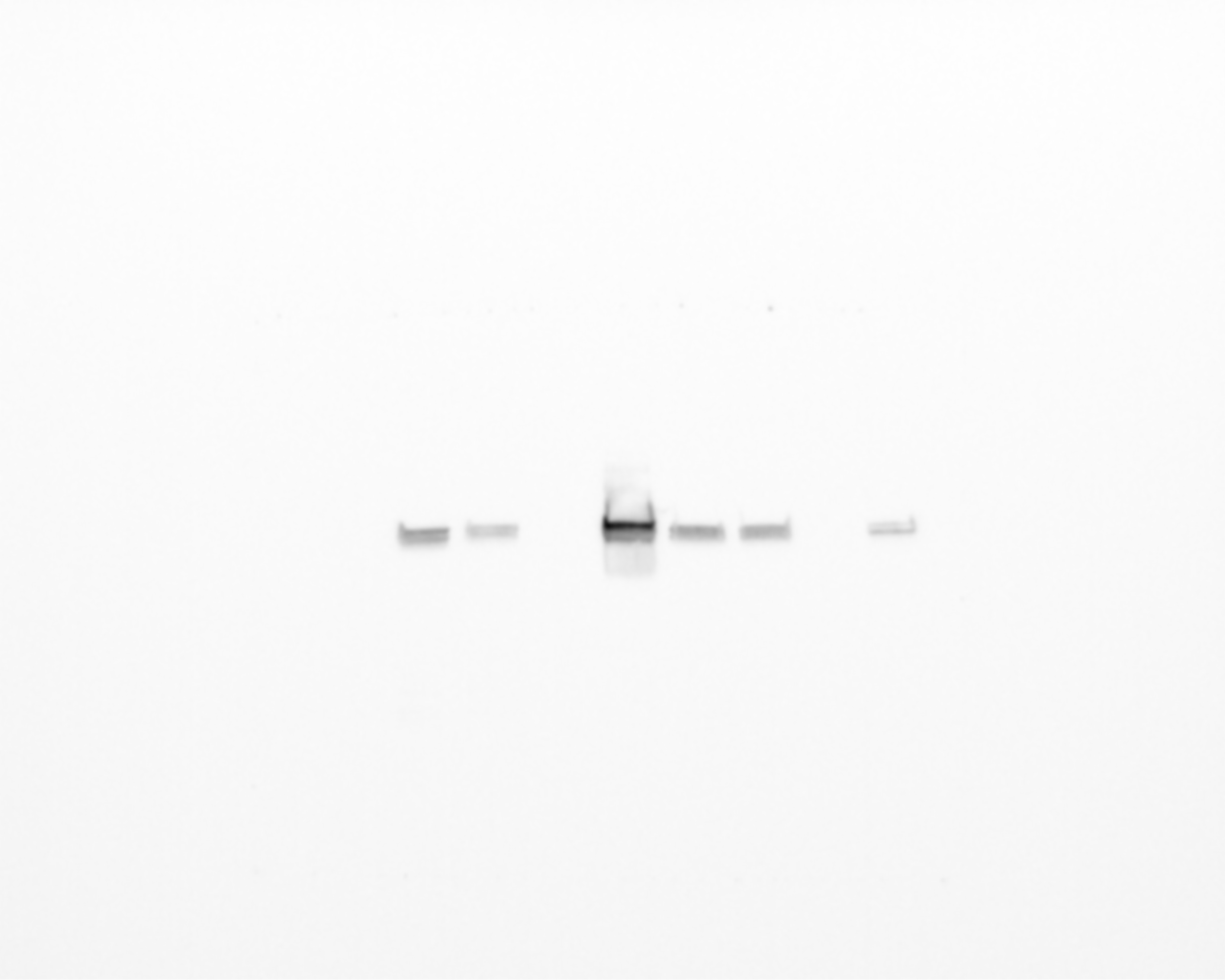

Supplement: Figure 6—source data 2. [file elife-84477-fig6-data2.zip › Figure 6-source data 2/SARS_CoV_2_Orf3a_wt_and_LC_mut_pulldown_50ug_GFP_blot_only.tif]

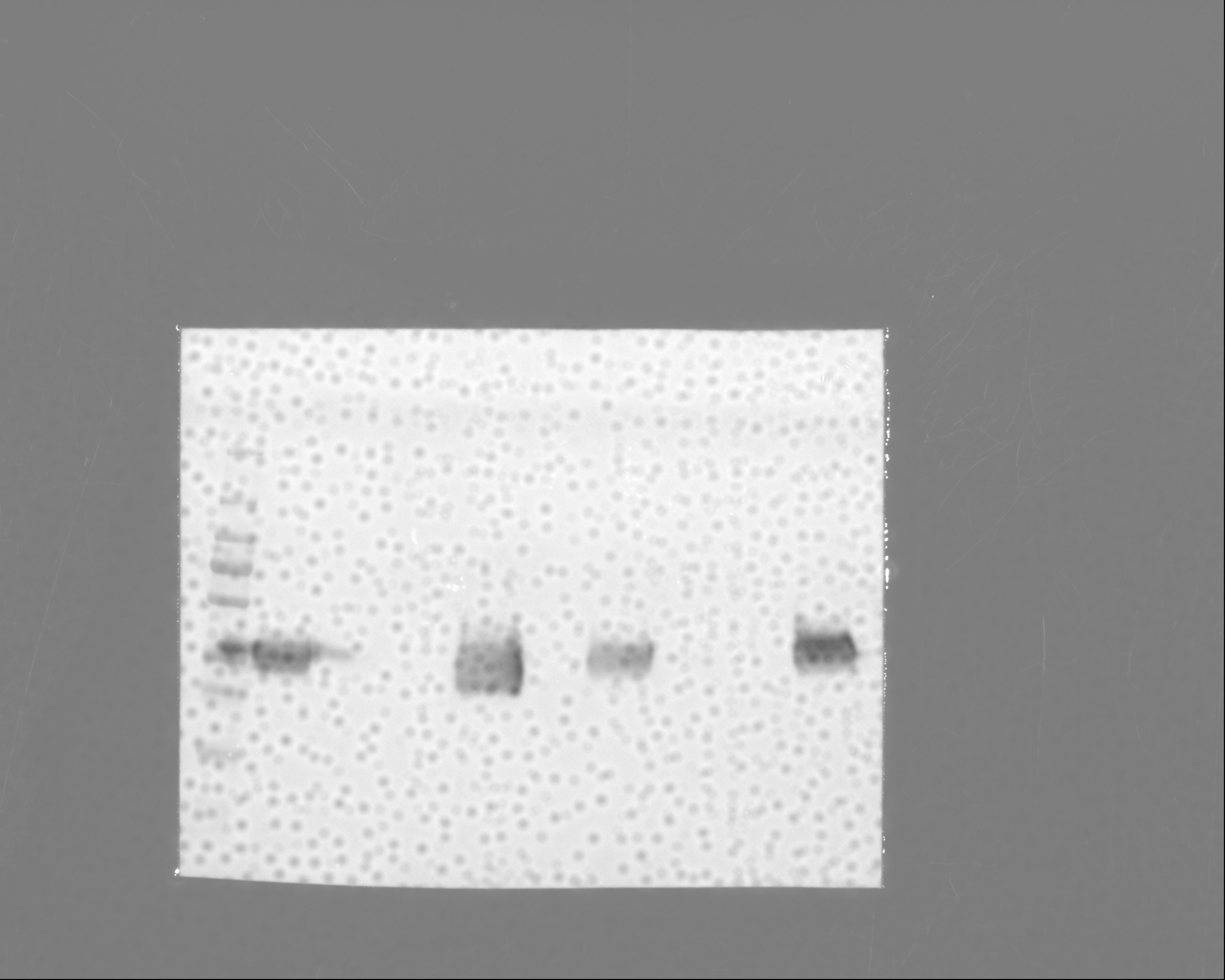

Supplement: Figure 6—source data 2. [file elife-84477-fig6-data2.zip › Figure 6-source data 2/SARS_CoV_1_Orf3a_wt_and_LC_mut_pulldown_50ug_strep_blot_standardsmerged.tif]

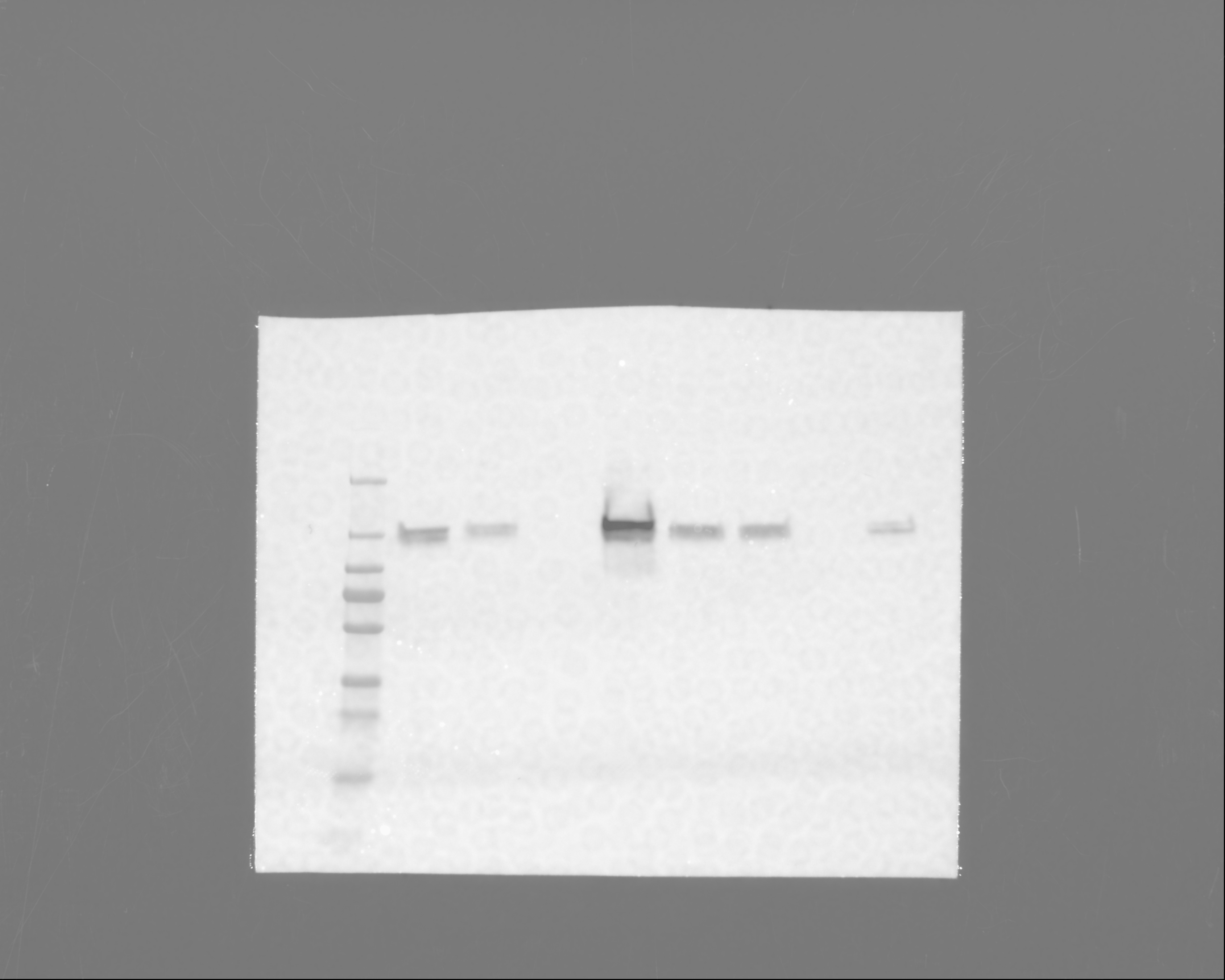

Supplement: Figure 6—source data 2. [file elife-84477-fig6-data2.zip › Figure 6-source data 2/SARS_CoV_2_Orf3a_wt_and_LC_mut_pulldown_50ug_GFP_blot_standardsmerged.tif]

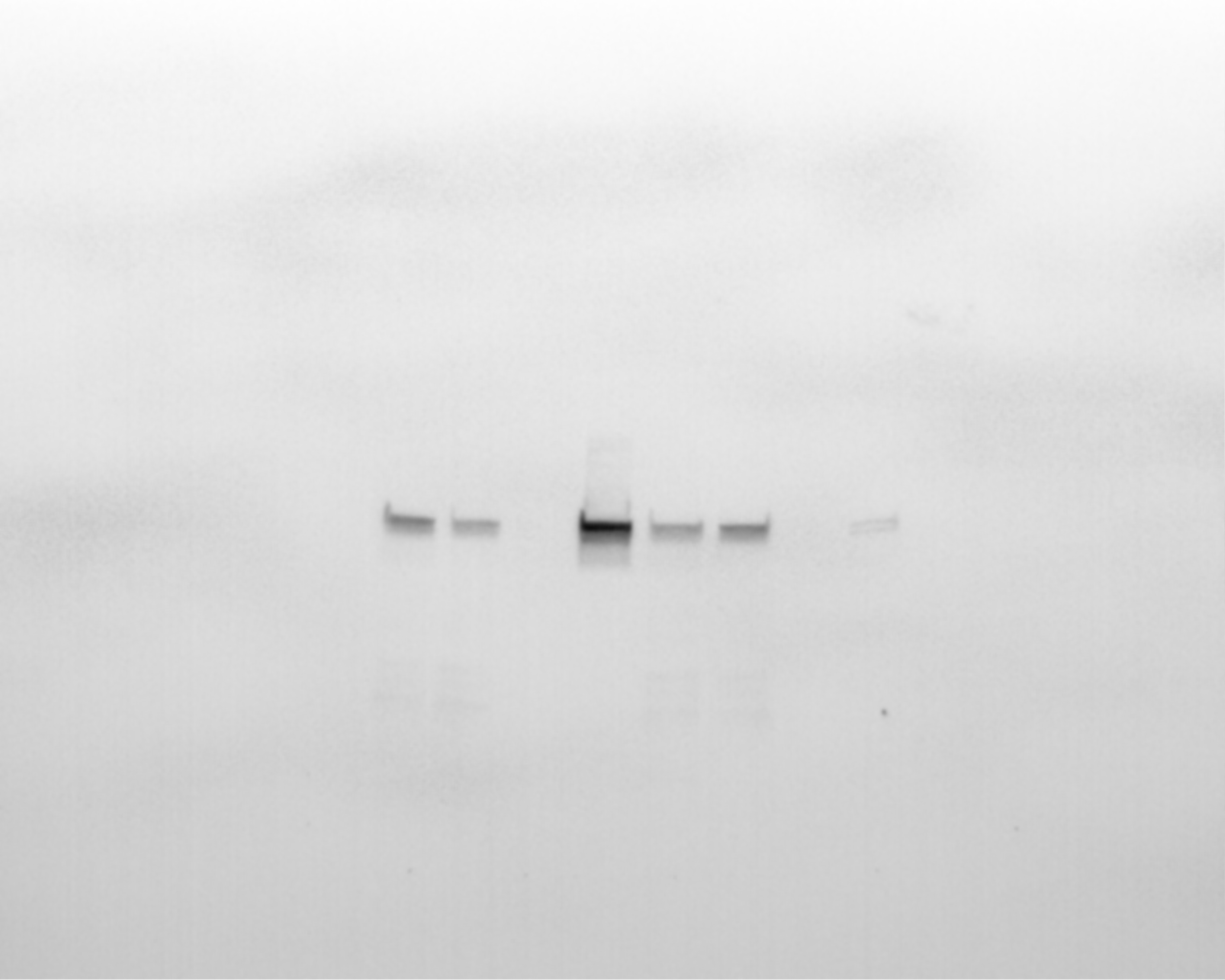

Supplement: Figure 6—source data 2. [file elife-84477-fig6-data2.zip › Figure 6-source data 2/SARS_CoV_2_Orf3a_wt_and_LC_mut_pulldown_5ug_GFP_blot_only.tif]
