# Supplementary material for: The SARS-CoV-2 accessory protein Orf3a is not an ion channel, but does interact with trafficking proteins
Source: eLife. 2023 Jan 25;12:e84477. doi: 10.7554/eLife.84477 (PMC9910834; doi:10.7554/eLife.84477)
Supplement: Supplementary file 1. [file elife-84477-supp1.docx]

**Supplementary File 1 | Cryo-EM data collection, refinement, and validation statistics**

|  | CoV-2 Orf3a, LE/Lyso MSP1D1 nanodisc  (EMDB-28538)  (PDB ID: 8EQJ) | CoV-2 Orf3a, PM  MSP1D1 nanodisc  (EMDB-28545)  (PDB ID: 8EQT) | CoV-2 Orf3a, LE/Lyso Saposin A nanodisc  (EMDB-28546)  (PDB ID: 8EQU) | CoV-1 Orf3a, LE/Lyso MSP1D1 nanodisc  EMDB-28544  (PDB ID: 8EQS) |
| --- | --- | --- | --- | --- |
| **Data collection** |  |  |  |  |
| Microscope | FEI Titan Krios |  |  |  |
| Camera | Gatan K3 |  |  |  |
| Energy filter | Gatan Image Filter  BioQuantum |  |  |  |
| Energy slit width | 20 eV |  |  |  |
| Spherical aberration corrector | to ~0.01 mm |  |  |  |
| Voltage (keV) | 300 |  |  |  |
| Calibrated magnification | x 59,242 |  |  |  |
| Electron exposure (e-/Å^2^) | 50 |  |  |  |
| Defocus range (μm) | -0.8 to -2.0 |  |  |  |
| Pixel size [super resolution] (Å) | 0.844 [0.422] |  |  |  |
|  |  |  |  |  |
| **Data processing** |  |  |  |  |
| Software | Relion 3.0  cryoSPARC 3.0 | Relion 3.1  cryoSPARC 3.0 | Relion 3.1  cryoSPARC 3.0 | Relion 3.1  cryoSPARC 3.0 |
| Symmetry imposed | C2 | C2 | C2 | C2 |
| Initial particle images (no.) | 7,135,081 | 8,567,984 | 11,247,076 | 7,433,020 |
| Final particle images (no.) | 178,997 | 125,625 | 135,280 | 162,607 |
| Overall map resolution (Å)  *FSC threshold 0.143* | 3.0 | 3.4 | 2.8 | 3.1 |
| Map sharpening *B* factor (Å^2^) | -50 | -50 | -50 | -50 |
|  |  |  |  |  |
| **Model building and refinement** |  |  |  |  |
| Software | Coot 0.9  Phenix 1.19 real-  space-refine | Coot 0.9  Phenix 1.19 real-  space-refine | Coot 0.9  Phenix 1.19 real-  space-refine | Coot 0.9  Phenix 1.19 real-  space-refine |
| Initial model used (PDB code) | 7KJR | 7KJR | 7KJR | 7KJR |
| Model resolution (Å)  *FSC threshold 0.5* | 3.1 | 3.5 | 2.9 | 3.2 |
| Model composition |  |  |  |  |
| Non-hydrogen atoms | 3240 | 3240 | 5766 | 3702 |
| Protein residues | 382 | 382 | 858 | 442 |
| Ligands | 4 | 4 | 2 | 2 |
| B factors (Å^2^) |  |  |  |  |
| Protein | 87.93 | 111.38 | 61.85 | 89.48 |
| Ligand | 136.73 | 176.82 | 101.32 | 133.54 |
| R.m.s. deviations |  |  |  |  |
| Bond length (Å) | 0.002 | 0.005 | 0.003 | 0.005 |
| Bond angle (**°)** | 0.516 | 0.418 | 0.523 | 0.868 |
| Validation |  |  |  |  |
| Molprobity score | 1.61 | 1.45 | 1.49 | 2.79 |
| Clashscore | 9.00 | 7.94 | 6.72 | 10.55 |
| Poor rotamers (%) | 0 | 0 | 0 | 0 |
| Ramachandran plot |  |  |  |  |
| Favored (%) | 97.34 | 97.86 | 97.37 | 97.21 |
| Unfavored (%) | 2.66 | 2.14 | 2.63 | 2.79 |
| Disallowed (%) | 0.0 | 0.0 | 0.0 | 0.0 |
